# Requirements for Achieving Self-Healing at Low/Room Temperature in Polymers

**DOI:** 10.1021/acs.macromol.5c01424

**Published:** 2025-10-11

**Authors:** Kanyarat Mantala, Daniel Crespy

**Affiliations:** Department of Materials Science and Engineering, School of Molecular Science and Engineering, 423058 Vidyasirimedhi Institute of Science and Technology (VISTEC), Rayong 21210, Thailand

## Abstract

Low-temperature self-healing polymers are crucial, as many real-world damage events occur in environments where external heating is impractical or energy-inefficient. However, achieving effective self-healing at these temperatures remains a significant challenge due to the restricted polymer chain mobility. To tackle this challenge, strategies have been investigated, such as modulating the strength of reversible chemical bonds; however, these approaches alone are often inadequate. In this Perspective, we comprehensively examine the factors influencing polymer chain mobility under low and ambient temperatures. We focus on optimizing material design to balance mechanical strength and healing performance, considering factors such as polymers with low glass transition temperatures, different types of polymers, branched to hyperbranched architectures, the role of shape-memory effects, and the facilitative impact of solvents. These insights provide a foundation for designing self-healing polymers tailored to specific application demands. Furthermore, we outline key considerations in synthetic design, molecular mobility, healing time, mechanical properties, and other functional properties, such as hydrophobicity and impedance modulus, as well as perspectives for creating materials that effectively self-heal at low or room temperatures.

## Graphical abstract

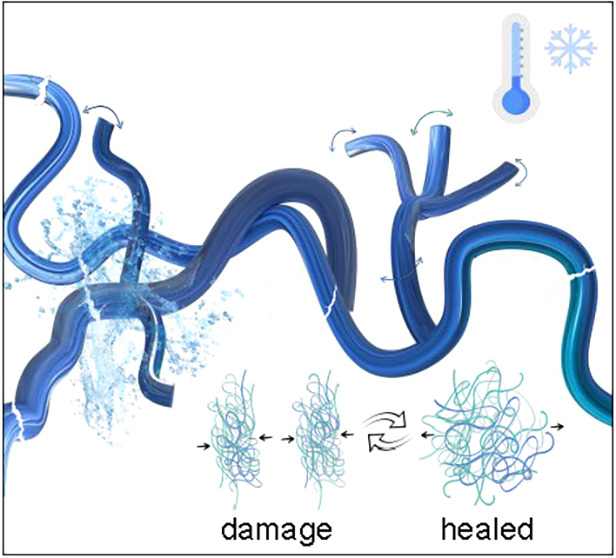

## 1 Introduction

Self-healing is an innate property of living organisms. The role of self-healing is to protect and maintain life and biological functions after external biological, chemical, or physical injuries.^1^ In mammals, blood cells or mobile cells serve to repair mechanical damage by blood-clotting cascade, followed by tissue regeneration.^2^ Plant cells, on the other hand, are immobile because cells are encapsulated within the cell wall. Thus, tissue regeneration in plants relies on oriented cell division and cell expansion to heal wounds.^3^ In contrast, most artificial or engineered materials do not have the ability to self-repair, potentially causing materials failure after mechanical damage, corrosion, or under fatigue. Such deteriorations can lead to economic loss and sometimes loss of human lives.^4^ To overcome this issue, scientists have tentatively mimicked some features of biological healing with polymer materials.^5–16^ The mechanism of extrinsic self-healing materials relies on dispersed capsules or vascular networks containing healing agents (monomers, initiators, or catalysts) that are mixed upon their fracture, leading to the formation of a new polymer that repair the damaged polymer.^17–20^ However, extrinsic self-healing materials cannot heal several times at the same location. On the contrary, intrinsic self-healing allows a damaged polymer to heal several times at the same location.^21,22^ Many designs of intrinsic self-healing polymers require an external stimulus such as light,^23–26^ heat,^27–31^ pressure,^32^ or the presence of solvent^33^ for imparting enough energy or mobility to the polymer materials to enable a significant chemical or physical change. This approach is reliable, but the use of external stimuli for self-healing can lead to detrimental side effects such as discoloring, crazing, bending, cracking, and swelling, which can impact the integrity of materials.^34,35^ Therefore, one of the foci of current research in the field of self-healing materials is to develop intrinsic self-healing materials that do not require additional external energy so that a truly autonomous healing becomes possible. The healing would then proceed at room or low temperatures, which is challenging. Indeed, at low temperatures, polymer chain dynamics are slow, which hinders the self-healing capability. The strategy for producing materials capable of healing at low temperatures relies on dynamic supramolecular interactions within the polymer matrix, such as ionic interactions,^36–38^ metal–ligand coordination,^39–41^ and hydrogen bonding,^42–44^ or on the presence of reversible covalent bonds.^45–47^ In addition, the high mobility of polymer chains in hydrogels^14,37,48–52^ and certain elastomers^36,41,43–45,53,54^ is favorable for imparting self-healing properties at room temperature. Self-healing in polymers can be broadly categorized into diffusion/mobility-controlled healing and reaction/bond exchange-controlled limited healing.^55,56^ In diffusion-controlled systems, healing primarily relies on the mobility of polymer chains across damaged interfaces. This regime typically requires sufficient segmental motion, which is favored in soft or amorphous domains and at temperatures above the glass transition temperature. This system relies on physical interactions such as van der Waals forces, ionic interactions, and loosely bound supramolecular motifs. In contrast, reaction-controlled healing is limited not by chain mobility but by the kinetics of bond exchange or reformation. These systems may involve dynamic covalent bonds such as disulfide bond exchange, imine exchange, or transesterification, as well as fast supramolecular interactions such as hydrogen bonding and metal–ligand coordination. Self-healing in glassy or highly cross-linked networks can proceed efficiently under low-mobility conditions when the bond exchange dynamics are sufficiently rapid. The classification into diffusion- or reaction-controlled regimes is governed more by the dominant kinetic process of either chain mobility or bond exchange than by the specific type of bonding involved. Many self-healing materials exhibit features of both regimes. However, identifying the dominant rate-limiting step is crucial for guiding the design of polymers tailored to specific healing conditions and performance requirements. This Perspective mainly focuses on diffusion-controlled healing.

Herein, we discuss the currently available pathways for the fabrication of polymer materials that can heal at low/room temperatures. To ensure consistency throughout this Perspective, we define the performance window as the temperature range within which self-healing behavior and functional performance are evaluated. Because the meaning of low and room temperature can differ between fields, we explicitly define the temperature range used in this perspective as −40 °C to 36 °C. Room temperature is defined as ranging from 20 °C to 36 °C based on the literature reviewed in this perspective, encompassing indoor and ambient conditions relevant to practical applications such as consumer electronics, structural coatings, and wearable devices. Low temperature refers to conditions below 20 °C, extending down to −40 °C. This range is based on the literature reviewed in this perspective and was selected to encompass subzero environments relevant to real-world, unheated operating conditions. Defining this window facilitates fair comparisons across the studies. We first describe the approaches that do not require chemical reactions and the presence of additional molecules. Then, we focus on materials healing in the presence of solvents.

## 2 Models of Chain Mobility Relying on Polymer Physics

Chain mobility, a critical factor in diffusion-controlled healing, can be understood through classical polymer dynamics models. In unentangled polymer systems, chain relaxation follows Rouse dynamics, where each segment moves independently, and the relaxation time scales with the square of the degree of polymerization (τ ∝ *N* ^2^).^57^ For entangled polymers, the reptation model is more applicable, describing chain motion as constrained within a hypothetical tube formed by the surrounding chains. In this case, the reptation time scales as τ ∝ *N* ^3^, reflecting significantly slower dynamics with increasing molecular weight.^58^ Polymers containing reversible or transient associations, such as hydrogen bonding, metal–ligand interactions, or dynamic covalent linkages, chain motion can be described by sticky-Rouse or sticky reptation models.^59,60^ These models introduce additional frictional effects due to temporary stickers with overall relaxation times influenced by both chain length and the lifetime or density of these dynamic interactions. A schematic scaling relation for such systems may be expressed as τ ∝ N^2^ + τ_sticker_, where τ_sticker_ represents the relaxation time for reversible bonds (Table 1). Incorporating these scaling concepts helps to clarify how molecular parameters such as chain length, entanglement density, and reversible bonding kinetics govern the time scales of self-healing. This polymer physics framework allows for a more mechanistic understanding of chain mobility and provides a valuable guide for designing materials with targeted healing behaviors.

**Table 1:** Summary of Polymer Models, Scaling of Relaxation Time, and Governing Mechanisms for Rotatation, Reptation, and Sticky-Rouse

model	type	scaling of relaxation time	governing mechanism
Rouse	unentangled polymer chains	τ ∝ *N* ^2^	segmental motion in dilute state
reptation	entangled polymer chains	τ ∝ *N* ^3^	chain slithering in entanglement tube
sticky-Rouse	polymer chains with reversible bonds	τ ∝ *N* ^2^ + τ_sticker_	segmental motion + dynamic bonding

## 3 Structure–Property Relationships of Self-Healing Polymers

A comprehensive understanding of the structure–property relationships in self-healing polymers is critical for the rational design of materials that can autonomously repair damage under low/room temperatures while maintaining a certain mechanical performance. Nonhealable polymers were endowed with a self-healing functionality through a blending approach.^61^ The self-healable poly­(ether thiourea) and nonhealable poly­(octamethylene thiourea) were synthesized via step-growth polycondensation between 1,1′-thiocarbonyldiimidazole and diamine monomers, namely, 1,2-bis­(2-aminoethoxy)­ethane and 1,8-diaminooctane, respectively (Figure 1a_1_). These polymers were subsequently combined by solution blending. Damaged interfaces with a diameter of 1 mm in polymer blends containing 20 mol % of poly­(ether thiourea) exhibited restored mechanical integrity, achieving a stress at break of 48 MPa at 32 °C under compression at 1 MPa for 1 h (Figure 1a_2_). The self-healing mechanism was attributed to hydrogen bond exchange and nanoscale phase separation, which enabled a sufficient mobility. Beyond hydrogen bond-driven healing in phase-separated blends, Urban et al. demonstrated that specific monomer sequences alone without dynamic bonding or phase separation can facilitate self-healing via interchain van der Waals interactions.^62^ In their study, self-healing copolymers of methyl methacrylate and *n*-butyl acrylate exhibited healing behavior that was highly dependent on the composition and chain topology. Copolymers were synthesized by atom transfer radical copolymerization of methyl methacrylate and *n*-butyl acrylate with molar ratios ranging from 30:70 to 70:30 (Figure 1b_1_). After damage, copolymers with methyl methacrylate to *n*-butyl acrylate ratios between 45:55 and 50:50 exhibited 90–100% recovery in tensile strain of ∼550% and stress of ∼8.6 MPa within 14 h without external assistance (Figure 1b_2_,b_4_,b_5_). These copolymers adopted extended helix-like conformations, resulting in a high cohesive energy density of 1.96 × 10^5^ kJ/m^3^ and enabling ambient self-repair driven by van der Waals interactions (Figure 1b_3_). Although the sequence was not strictly controlled, the copolymer chain predominantly featured an alternating-rich arrangement. Sequence control was employed as a strategy to achieve more predictable structure–property relationships.^63^ An alternating copolymer of methyl methacrylate and *n*-butyl acrylate was synthesized by copolymerizing *n*-butyl acrylate with a sterically hindered 3,5,6-trichlorosalicylic acid–based methacrylate bearing an adamantyl group. Subsequent postpolymerization transesterification was performed to replace the pendant ester group with a methyl group, producing a regular alternating arrangement of methyl methacrylate and *n*-butyl acrylate units along polymer chains (Figure 1c_1_). The adamantyl-containing methacrylate monomer exhibited low homopolymerization reactivity but readily formed an alternating copolymer with an *n*-butyl acrylate. The resulting copolymer demonstrated rapid self-healing at ∼25 °C, fully restoring a Young’s modulus of 8 MPa within 15 min (Figure 1c_2_). This rapid and efficient healing behavior was attributed to a highly viscoelastic flow and the uniform monomer arrangement, which facilitated strong “key-and-lock” interactions between alternating segments (Figure 1c_3_). Collectively, these studies highlight how different structural strategies, i.e., phase-separated blends, alternating sequences, or carefully tuned copolymer topologies, govern the dynamic interactions that enable self-healing polymers at low/room temperatures.

**Figure 1:**
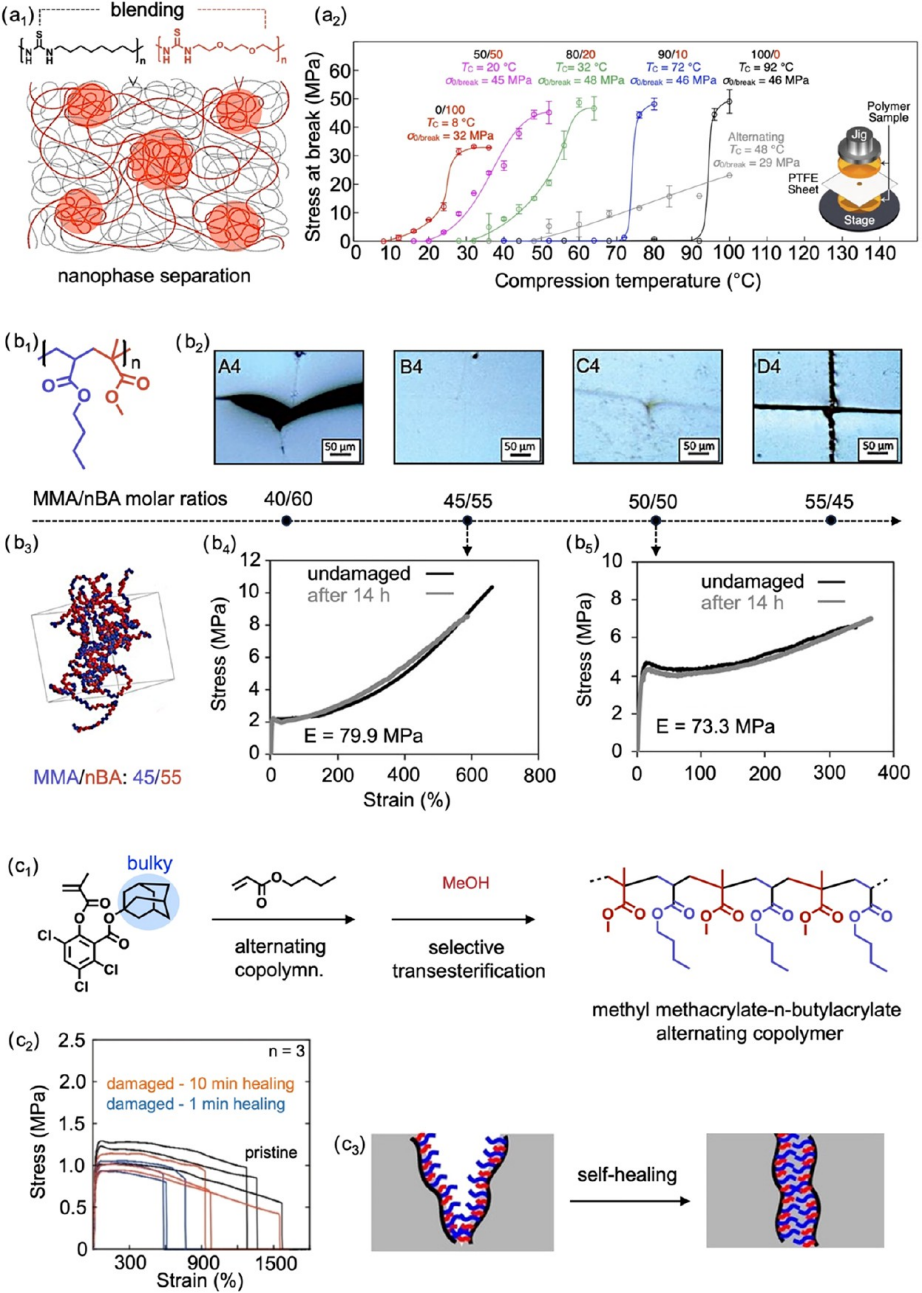
(a_1_) Blending of nonhealable poly­(octamethylene thiourea) with room-temperature self-healable poly­(ether thiourea), exhibiting nanophase-separated structure. Adapted with permission.^61^ Copyright 2022, Wiley. (a_2_) Healing behaviors of poly­(octamethylene thiourea)/poly­(ether thiourea) blends at varying polymer molar ratios: 100/0 (black), 90/10 (blue), 80/20 (green), 50/50 (pink), and 0/100 (red), after 1 h of compression at designated temperatures. (b_1_) Chemical structure of methyl methacrylate and *n*-butyl acrylate alternating-rich copolymer. (b_2_) Optical images of damage poly­(methyl methacrylate/*n*-butyl acrylate) with varying molar ratios: 40/60, 45/55, 50/50, and 55/45. Adapted with permission.^62^ Copyright 2018, Science. (b_3_) Morphology of an alternating-rich poly­(methyl methacrylate/*n*-butyl acrylate) copolymer with a molar ratio of 45/55. Tensile stress–strain curves of poly­(methyl methacrylate/*n*-butyl acrylate) copolymer with a molar ratio of 45/55 (b_4_) and 50/50 (b_5_) before damage and after healing for 14 h at ∼25 °C. (c_1_) Reaction scheme of poly­(methyl methacrylate/*n*-butyl acrylate) alternating copolymer. Adapted with permission.^63^ Copyright 2023, Wiley. (c_2_) Tensile stress–strain curves of poly­(methyl methacrylate/*n*-butyl acrylate) alternating copolymer after healing for 1 and 10 min at ∼25 °C. (c_3_) Schematic of the self-healing mechanism based on viscoelastic flow and lock-and-key interactions between alternating sequence segments.

## 4 Vitrimers-Based Self-Healing Polymers

Vitrimers represent a distinct and increasingly prominent class of self-healing materials, characterized by dynamic covalent networks that undergo associative bond exchange.^64^ In this mechanism, bond cleavage and bond formation occur simultaneously, allowing the network to rearrange while maintaining a constant cross-link density. Physicochemical approaches, in contrast, rely on dissociative mechanisms. Dissociative mechanisms involve bond cleavage prior to new bond formation, which can lead to transient reductions in cross-link density and compromise structural integrity. Vitrimers avoid this limitation by maintaining a constant cross-link density during rearrangement, preserving structural integrity and offering enhanced thermal and chemical stability. Various dynamic bonds inspired by vitrimer chemistry have been utilized for the recycling and self-healing of polymers, including vinylogous urethane,^65^ dioxaborolane,^66^ and S–S exchange.^67^ The vinylogous urethane vitrimer was synthesized by transamination of poly­(ethylene glycol)-based acetoacetate with tris­(2-aminoethyl)­amine in the presence of potassium hexafluorophosphate.^65^ The full cut vitrimer was healed at 25 °C for 8 h, recovering ∼50% of the original tensile strength of ∼1.5 MPa. This efficient self-healing behavior was attributed to ion-assisted catalysis of the vinylogous urethane transamination, which facilitated a topological rearrangement with an activation energy of ∼103 kJ mol^–1^. However, incomplete mechanical recovery suggests limitations in bond reformation or polymer mobility.

To enhance healing efficiency, an epoxy-based vitrimer-like material was developed by incorporating both aromatic disulfide and hydrogen bonding.^67^ The epoxy-based material was synthesized by an epoxy ring opening reaction between bisphenol A diglycidyl ether, poly­(propylene glycol)­diglycidyl ether, and diglycidyl 1,2-cyclohexanedicarboxylate, using 2-aminophenyl disulfide as the curing agent. The damaged vitrimer recovered 80% of its original tensile strength of ∼1 MPa after 96 h at 25 °C without the need for external stimuli. The healing behavior was attributed to *T*_g_ ∼ 20 °C below room temperature, which facilitated chain mobility and enabled reversible aromatic disulfide exchange in combination with hydrogen bonding.

A similar subambient healing approach was employed using a silicone vitrimer based on dioxaborolane exchange.^66^ The silicone vitrimer was first synthesized through photoinitiated thiol–ene coupling between vinyl-terminated polydimethylsiloxane and thiol-functionalized dioxaborolane. The network was then reinforced with silica nanofillers of 23 wt % to improve stiffness and creep resistance. Damaged vitrimer exhibited ∼100% mechanical recovery of ∼1.4 MPa of tensile strength at 25 °C for 62 days. The healing mechanism was based on the high mobility of the silicone chains, which promoted the dioxaborolane exchange with an activation energy of 8.5 kJ mol^–1^, enabling room-temperature healing without external catalysts. These studies highlight the potential of vitrimers to bridge the performance gap between dynamic covalent and supramolecular nanomaterials in applications requiring autonomous and low-energy healing.

## 5 Self-Healing through High Mobility in Polymers

Physical self-healing relies on molecular interdiffusion, induced by Brownian motion, chain segment motion, or entropy-driven movement of molecules with and without additional stimuli.^2^ Crack healing of polymer materials can be divided into 5 stages which are segmental surface rearrangements, surface approach, wetting, diffusion, and randomization.^68^ Topography and roughness of the surfaces, chain-end distribution, molecular weight, and molecular weight distribution are factors significantly affecting surface rearrangements. Physical and/or chemical self-healing with or without solvent occurs through contact with cracked surfaces. The damaged surfaces need hence to wet each other to form an interface. The diffusion process is the most critical stage for the recovery of mechanical strength, because the fractal random walk of polymer chains close to the surface allows entanglements of mobile chains. Therefore, local mobility and diffusion rates at the interfacial areas are essential for self-healing. The initial crack interface will then vanish during the randomization process.

### 5.1 Polysiloxanes

Self-healing materials based on poly­(siloxanes) were employed to produce electronic skin, wearable electronic devices, and artificial muscles. The high flexibility of siloxane linkages^69–71^ and the relatively weak interaction between the polydimethylsiloxane (PDMS) chains lead to a low glass-transition temperature (*T*_g_) of ca. −123 °C.^47,72,73^ This low *T*_g_ allows long-range segmental motions at subzero temperatures, which can be exploited in PDMS elastomers or in other polymers containing PDMS segments. Polydimethylsiloxane does not inherently possess efficient self-healing properties unless specifically designed to incorporate this function. Self-healing polydimethylsiloxanes were designed to display a combination of dynamic covalent and noncovalent bonds such as imine,^74,75^ hydrogen bonds,^76^ aliphatic or aromatic disulfide/hydrogen bonds,^47,77,78^ metal-ligand coordination/hydrogen bonds,^79,80^ and boronate ester/hydrogen bonds.^81^ Poly­(urea) was engineered with long hard segments to form elastomers (Figure 2a) and synthesized through a two-step polyaddition process.^77^ The long hard segment was formed by the reaction between bis­(trifluoromethyl)­benzidine and 4-aminophenyl disulfide, along with a mixture of diisocyanates, including diisothiocyanate and hexamethylene diisocyanate. Subsequently, diamine-terminated polydimethylsiloxane was incorporated for chain expansion. The full-cut sample was able to recover 56% of its tensile strength at break (∼1.4 MPa relative to the original ∼2.5 MPa) after healing in air at 30 °C for 24 h and showed a healing efficiency of 82%, reaching a tensile strength of ∼2.0 MPa after healing in sheep blood at 35 °C for 24 h. The self-healing mechanism relied on the high mobility of the polymer chains due to the low *T*_g_ of −120 °C from the PDMS soft segment which promoted dynamic hydrogen and disulfide bonds exchange. Similar self-healing times and higher healing efficiencies for polydimethylsiloxane-based poly­(urea-urethane) containing spiropyran and terpyridine through a polyaddition reaction.^79^ Subsequently, a solution of terbium chloride hexahydrate was mixed with a solution of poly­(urea-urethane) to form metal–ligand coordination bonds. Bisected samples were able to fully recover their toughness, achieving 100% healing efficiency with a tensile strength of ∼0.25 MPa after healing for 24 h at 25 °C, owing to the dynamic nature of hydrogen bonding and the metal–ligand coordination interactions (Figure 2b). Diffusion and mobility of the polymer chains are enhanced by their noncrystalline and loose structure, and the low *T*_g_ (<−80 °C), which promoted self-healing (Figure 2c).

**Figure 2:**
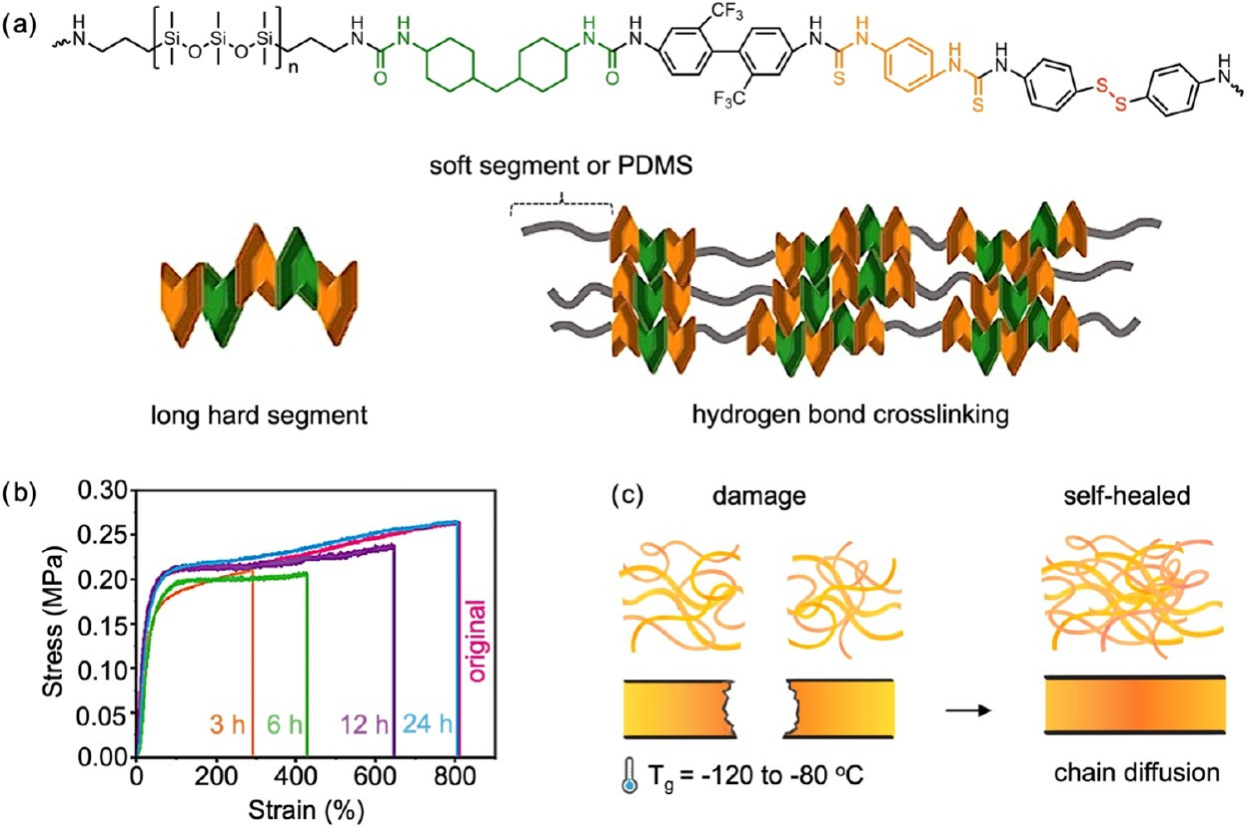
(a) Schematic illustration of a polydimethylsiloxane-based polyurea. Adapted with permission.^77^ Copyright 2024, Elsevier. (b) Tensile stress–strain curves of fractured photochromic polymers which are healed for various durations. Adapted with permission.^79^ Copyright 2024, Wiley. (c) Proposed self-healing mechanism of the polydimethylsiloxane-based polymer.

To study self-healing materials at subzero temperatures, an elastomer of polydimethylsiloxan-containing imine bonds (I-PDMS) was prepared through a Schiff-base reaction between benzene-1,3,5-tricarbaldehyde and aminopropyl-terminated PDMS (Figure 3a).^74,74^ SiO_2_ was then introduced into the polymer in a tetrahydrofuran solution. The I-PDMS/SiO_2_ coatings after O_2_ plasma etching could recover their superhydrophobicity, exhibiting a water contact angle of 162 ± 1° and a sliding angle of 1° ± 1° after 5 h in vacuum at 25 °C, 28 h in water at 25 °C, and 72 h at −30 °C. In addition, the surface energy of the healed I-PDMS/SiO_2_ coatings after 5 h at 25 °C was the same as that of the pristine sample (25 ± 1 mJ m^–2^), indicating a self-healing of the hydrophobicity property (Figure 3b). The full recovery of superhydrophobicity resulted from dynamic imine bonds, where benzene-1,3,5-tricarbaldehyde and aminopropyl-terminated PDMS diffused to the damaged area and reformed, enabling self-healing (Figure 3c). Additionally, the low *T*_g_ of polydimethylsiloxane (below −60 °C) facilitated mobility at subzero temperatures.

**Figure 3:**
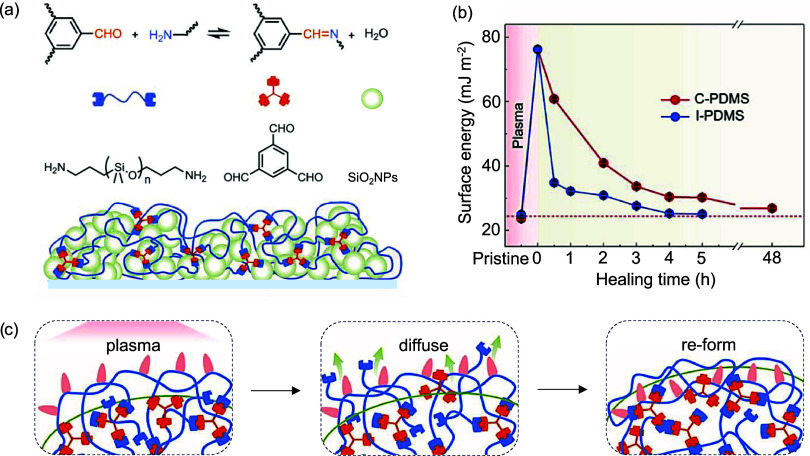
(a) Illustration of the fabrication process for polydimethylsiloxane coatings incorporating imine bonds and silicon dioxide nanoparticles (I-PDMS/SiO_2_). (b) Changes in surface energy of imine-based polydimethylsiloxane (I-PDMS) and cured polydimethylsiloxane (C-PDMS) films following plasma etching and subsequent healing over various durations. (c) Schematic of the self-healing mechanism of I-PDMS/SiO_2_ coatings. Plasma etching introduces polar groups, which are subsequently embedded, due to their higher surface energy, by newly formed I-PDMS through the diffusion and reaction of benzene-1,3,5-tricarbaldehyde and aminopropyl-terminated PDMS. Adapted with permission.^74^ Copyright 2024, Elsevier.

Guo et al. reported a polydimethylsiloxane-based poly­(urea-urethane) that achieved an even faster healing at lower temperature, down to −40 °C.^70^ Poly­(urea-urethane) elastomers were produced through the polyaddition reaction between α,ω-dihydroxyethylpropoxyl-PDMS (HO-PDMS–OH, *M*_n_ = 4600 g mol^–1^) and a diisocyanate, in the presence of 4,4′-dithioaniline and 4,4′-bis­(hydroxymethyl)-2,2′-bipyridine as chain extenders (Figure 4a). The fractured elastomer was mended at room temperature within 2 h, resulting in 93% healing efficiency with tensile strength of ∼0.074 MPa out of the original ∼0.08 MPa. At −40 °C, it exhibited ∼50% healing efficiency with a tensile strength of ∼0.04 MPa after 24 h, as calculated from tensile test measurements before and after healing. Healing at ultralow temperatures was possible due to a low *T*_g_ (<−50 °C) of the elastomer, yielding to sufficient re-entanglement of polymer chains at the fractured surface. Faster healing time with polysiloxane-based materials was achieved by Wang et al.^75^ Bis­(3-aminopropyl)-terminated polydimethylsiloxane (H_2_N-PDMS-NH_2_, *M*_n_ = 25,000 g mol^–1^) or poly­(propylene glycol)­bis­(2-aminopropyl ether) (H_2_N-PPG-NH_2_), *M*_n_ = 4000 g mol^–1^ (for preparing control sample) was reacted with 1,1′-biphenyl-3,3′,5,5′-tetracarbaldehyde by an aldimine polycondensation. Damaged PDMS- and PPG-based elastomers exhibited a healing efficiency from tensile measurements of 99% with tensile strength of 0.11 MPa within 60 s and 84% with tensile strength of 0.65 MPa out of an original 0.77 MPa within 12 h at 25 °C, respectively (Figure 4b). The fast self-healing performance was attributed to the long chains in the PDMS-based elastomer and consequent entanglement, which assisted effective dynamic imine exchange reactions within and between polymer chains. Tang et al. reported the fastest self-healing speed for polydimethylsiloxane-based materials.^81^ A poly­(dimethylsiloxane-dithiothreitol) block copolymer was synthesized by thiol–ene Michael polyaddition between vinyl-terminated PDMS and dithiothreitol, followed by the condensation of boric acid with the hydroxyl groups of the dithiothreitol units. The fully cut sample was completely mended at room temperature within 30 s without external stimuli. In addition, the damaged sample exhibited 96% recovery of the mechanical strength of ∼0.2 MPa after 2 min at −20 °C, attributed to the high mobility of polymer backbone and abundant hydrogen bonding, facilitating an immediate self-healing (Figure 4c). However, these healable PDMS-based elastomers displayed low ultimate tensile strength values (<2.5 MPa), potentially limiting their range of applications and hence motivating the search for polymers simultaneously displaying the contradictory high degree of mobility and high mechanical properties.

**Figure 4:**
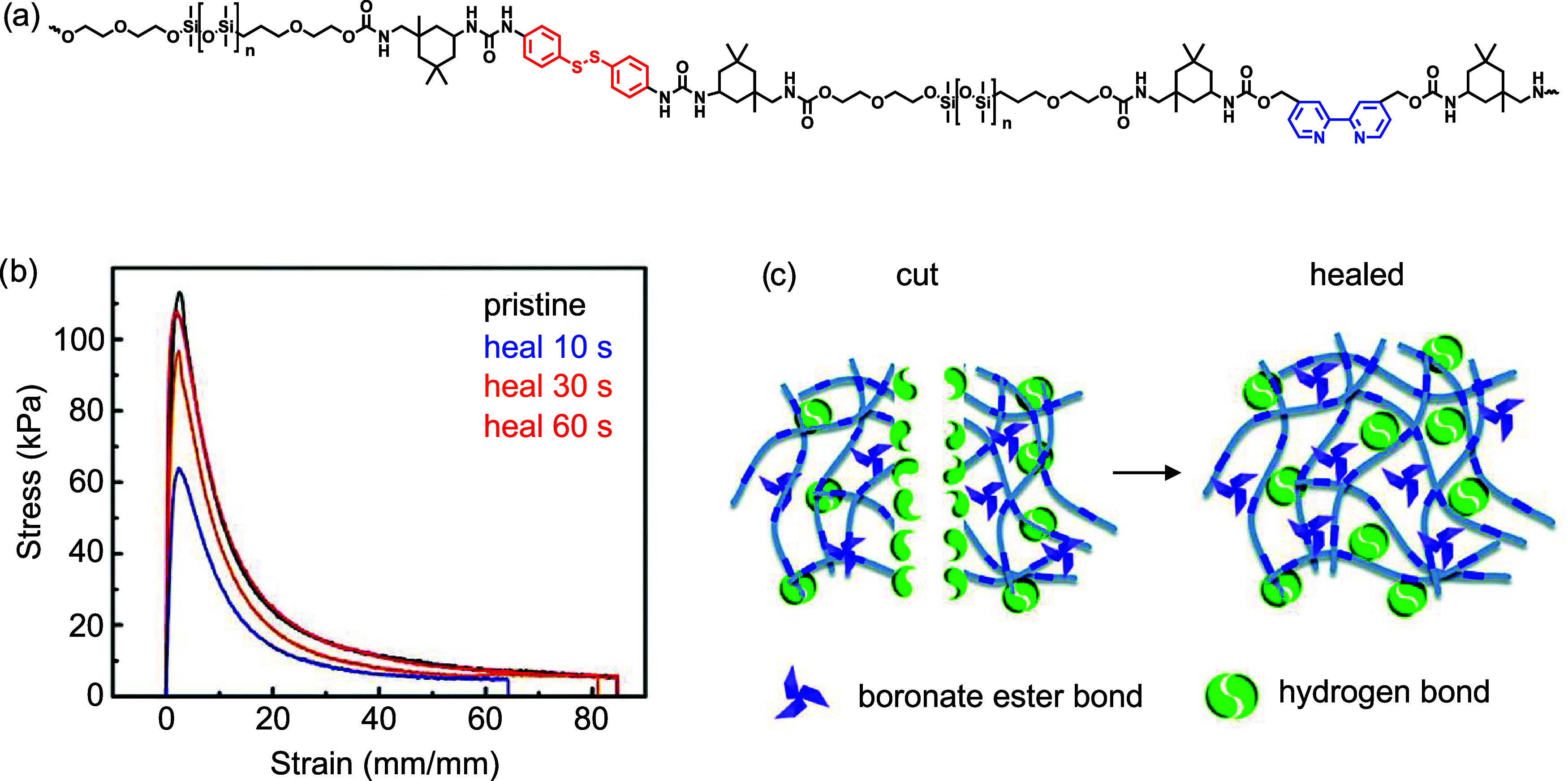
(a) Chemical structure of polydimethylsiloxane-based poly­(urea-urethane) containing dynamic aromatic disulfide and hydrogen bonds. Reproduced under terms of the CC-BY license.^70^ Copyright 2020, The Authors, published by Springer Nature. (b) Tensile stress-strain curves of self-healing polydimethylsiloxane with a tetra-functional biphenyl unit for 10, 30, and 60 s under 25 °C, 60 ± 5% RH. Adapted with permission.^75^ Copyright 2019, Royal Society of Chemistry. (c) Self-healing mechanism of a poly­(dimethylsiloxane-dithiothreitol) containing boronate ester bonds. Adapted with permission.^81^ Copyright 2022, Royal Society of Chemistry.

### 5.2 Polyacrylates with Long Side Chains

Polyacrylates bearing long alkyl side chains such as oligo­(ethylene glycol) or morpholine pendants can also exhibit rapid self-healing at room temperature. These flexible side chains enhance chain mobility, enable reversible interactions, and provide tunable mechanical properties, making them attractive candidates for room-temperature self-healing polymers.

Thus, a synthetic elastomer mimicking the stretchability, stiffness, and fatigue resistance of natural ligaments was prepared.^82^ Methacrylate-acrylate-based copolymer was synthesized by a photoinitiated free-radical polymerization of methyl methacrylate and di­(ethylene glycol) methyl ether acrylate in the presence of lithium bis­(trifluoromethanesulfonyl)­imide. The resulting material comprised soft, brush-like poly­(di­(ethylene glycol) methyl ether acrylate) segments and rigid poly­(methyl methacrylate) blocks, forming a hierarchical double-network reinforced by lithium-ether ion-dipole interactions and poly­(methyl methacrylate) nanodomains (Figure 5a_1_). The full cut elastomer was healed at 25 °C for 24 h, regaining ∼98% of its tensile stress of ∼0.32 MPa. Healing was driven by reformation of lithium-ether ion–dipole interactions and reaggregation of poly­(methyl methacrylate) domains (Figure 5a_2_).

**Figure 5:**
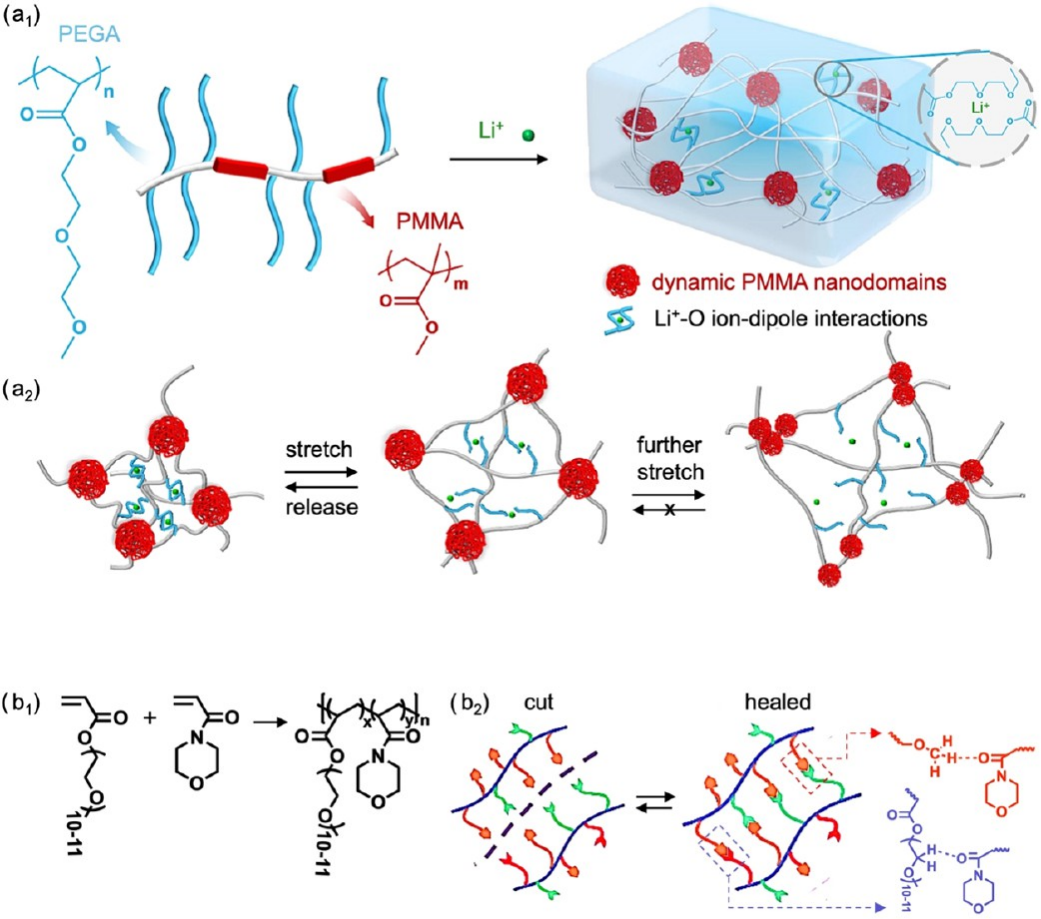
(a_1_) Chemical structure of poly­((diethylene glycol methyl ether acrylate)-*co*-methyl methacrylate) (PEGA-*co*-PMMA) in the presence of lithium bis­(trifluoromethanesulfonyl)­imide. Schematic representation of the double-cross-linked network formed via dynamic poly­(methyl methacrylate) and lithium–oxygen ion-dipole interactions. (a_2_) Conceptual model illustrating bond breakage and reformation during tensile stretching. At small strains, lithium–oxygen ion-dipole interactions dissociate easily and reform upon stress release. At larger strains, poly­(methyl methacrylate) nanodomains fragment into smaller domains, contributing to additional energy dissipation. Reproduced under terms of the CC-BY license.^82^ Copyright 2022, The Authors, published by Springer Nature. (b_1_) Reaction scheme of poly­((ethylene glycol) methyl ether acrylate-*co*-acryloylmorpholine). (b_2_) self-healing mechanism of the copolymer relying on dynamic hydrogen bonding. Adapted with permission.^88^ Copyright 2022, Elsevier.

In contrast, rapid self-healing at low temperature was enabled by an ionogel formed in a deep eutectic solvent.^83^ The ionogel was synthesized by a free-radical polymerization of acrylamide and methoxypoly­(ethylene glycol) methacrylate in a deep eutectic solvent composed of choline chloride and glycerol. Damaged ionogels were healed in 30 s at 25 °C and 10 min at −25 °C with 90% restoring tensile strength of 0.21 MPa and >85% efficiency, respectively. The rapid self-healing ability was driven by reversible hydrogen bonding between amide and ether groups, with the deep eutectic solvent matrix acting as a plasticizer to enhance chain mobility and reassociation at both room and subzero temperatures. However, the mechanical strength remained relatively limited.

In another study, a copolymer of poly­(ethylene glycol) methacrylate and 2-ureido-4­[1H]-pyrimidinone-functionalized hydroxyethyl methacrylate was prepared by free-radical polymerization.^84^ Polypyrrole was then incorporated via oxidative polymerization using ferric chloride as an oxidant. The full-cut copolymer achieved ∼100% recovery of tensile strength of ∼0.72 MPa and fully restored conductivity within 5 min at 25 °C. The healing ability was driven by reversible quadruple hydrogen bonding interactions between ureido-pyrimidinone units, enabling network reformation and restoration of conductivity at room temperature. However, its limited mechanical strength motivated the development of dual-dynamic networks with improved structural robustness.^85^ Poly­(methacrylic acid-*co*-oligo­(ethylene glycol) methacrylate) was synthesized by reversible addition–fragmentation chain transfer polymerization, followed by postfunctionalization with 4-benzaldehyde. The resulting copolymer was then cross-linked with ethylenediamine to produce imine bonds. The full cut polymer exhibited healing efficiency of 87% restoring tensile strength of >2.5 MPa after 40 min at 25 °C. The healing mechanism relied on synergistic hydrogen bonding and dynamic imine bond exchange, enabling both initial recovery and strengthening of network at room temperature. They later incorporated photoresponsive motifs to enable faster healing and light-driven actuation.^86^ The self-healable hydrogel was synthesized via reversible addition–fragmentation chain transfer polymerization of methacrylic acid and oligo­(ethylene glycol) methacrylate, followed by esterification with a benzyl imine-functionalized anthracene to introduce a light-sensitive and supramolecular functionality. The full cut hydrogel was healed autonomously at 35 °C, recovering ∼98% of its tensile strength, 3.67 MPa within 8 min. The healing was driven by synergistic π-π stacking interactions between anthracene groups and acid-ether hydrogen bonding, both of which were reversible and contributed to fast chain reassociation. Despite its excellent mechanical strength and rapid recovery, the system required moderately elevated temperatures to initiate healing.

To eliminate the need for elevated temperatures while maintaining rapid and efficient healing, Zhao et al. sought to overcome the trade-off between healing speed and mechanical strength in self-healing elastomers.^87^ They synthesized a copolymer using free-radical polymerization of poly­(ethylene glycol) methyl ether acrylate and *N*-acryloylmorpholine to form a hydrogen-bonded network (Figure 5b_1_). The completely cut elastomer exhibited 99% healing efficiency, recovering a tensile strength of 4.22 MPa within 40 s at 25 °C. The healing mechanism was based on multiple weak hydrogen bonds among methylene, methoxyl, and amide carbonyl groups, enabling rapid chain diffusion and reconnection (Figure 5b_2_).

Building on their earlier hydrogen-bonded system, the authors designed an anisotropic network to simultaneously enhance strength and retain rapid healing.^88^ The anisotropic hydrogel was synthesized via free-radical polymerization of poly­(ethylene glycol) methyl ether acrylate and *N*-acryloylmorpholine in water, initiated by potassium persulfate. The resulting hydrogel was air-dried under a 200% fixed uniaxial strain to form a hierarchically anisotropic network. The fully cut sample was healed autonomously at 25 °C, recovering 86% of its tensile strength, 7.3 MPa within 10 s. The rapid healing was enabled by weak hydrogen bonding between methylene groups and amide carbonyls, supported by chain alignment from the drying-induced anisotropic structure. The increased chain orientation slightly reduced healing efficiency compared to isotropic formulations due to limited polymer chain mobility.

To explore an alternative route to fast healing and strong mechanical properties, Xu et al. modulated nanoscale phase separation rather than relying on macroscopic chain alignment.^89^ The elastomer was synthesized via reversible addition–fragmentation chain transfer polymerization using poly­(ethylene glycol) methyl ether acrylate and *N*-acryloylmorpholine as monomers, with macro-chain transfer agent precursors formed by prepolymerizing *N*-acryloylmorpholine. By adjusting the molecular weight of the macro-chain transfer agent, they achieved tunable phase separation sizes from 15 to 9 nm. The fully cut samples were healed at 25 °C within 10 s, with healing efficiencies of 89%, 91.6%, and 92.7% and corresponding tensile strengths of 4.8, 6.9, and 7.8 MPa, respectively. Healing was enabled by reversible hydrogen bonding between amide groups, and the reduced domain size enhanced rebonding kinetics by increasing the local density of mobile chains. However, further reduction of the phase size below 9 nm was not achieved, leaving open the question of whether smaller domains might improve or hinder the performance.

To further enhance mechanical robustness while retaining fast healing, metal–ligand coordination was introduced into an anisotropic network based on the same monomer pair.^90^ The polymer was synthesized via free-radical copolymerization of poly­(ethylene glycol) methyl ether acrylate and *N*-acryloylmorpholine, initiated by potassium persulfate. After the anisotropic network was formed through drying under 200% strain, the material was further reinforced by immersing it in aqueous ZnCl_2_ to introduce metal–ligand coordination bonds. The damaged elastomer healed autonomously at 25 °C within 30 s, recovering ∼94.9% of its original tensile strength of 12.03 MPa. Healing was enabled by the synergistic interaction of reversible hydrogen bonds and strong Zn^2+^-carbonyl coordination bonds, which offered both rapid reassociation and structural reinforcement. However, excessive Zn^2+^ loading of ≥5 wt % reduced healing efficiency, likely due to limited chain mobility caused by overcross-linking.

Instead of relying on metal coordination, self-healing elastomers with both ultrafast recovery and high mechanical strength can be designed by leveraging chain entanglement and anisotropic structuring.^91^ The polymer was synthesized via free-radical copolymerization of poly­(ethylene glycol) methyl ether acrylate and *N*-acryloylmorpholine, using potassium persulfate and *N,N,N*′*,N*′-tetramethylethylenediamine as initiators. Chain entanglement was enhanced by increasing the monomer concentration and lowering the polymerization temperature, while the anisotropic structure was produced by drying the polymer under 200% fixed strain (Figure 6). The fully cut elastomer healed autonomously at 25 °C within 30 s, showing 18.4 MPa tensile strength and 86.2% healing efficiency. Healing was driven by reversible hydrogen bonding between ether and carbonyl groups, reinforced by topological chain entanglement and directional alignment. However, excessive entanglement or strain orientation reduced healing efficiency due to restricted chain mobility and fewer accessible bonding sites.

**Figure 6:**
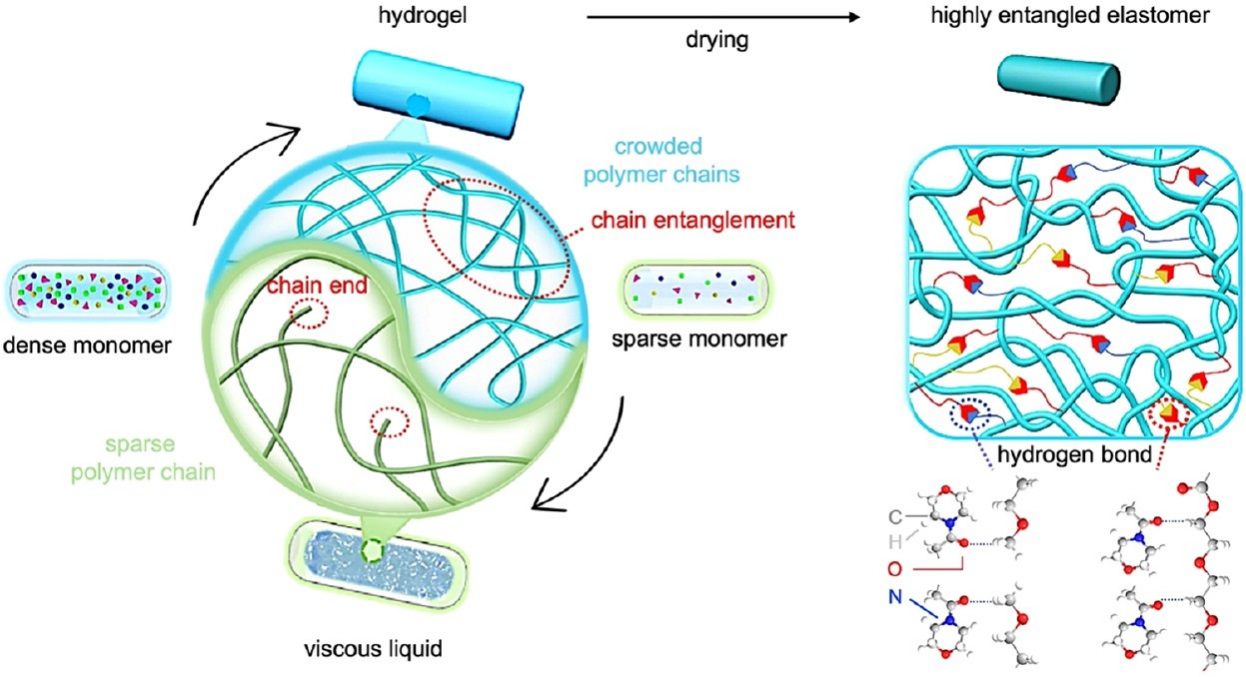
Schematic illustration of the fabrication process of a highly entangled poly­((ethylene glycol) methyl ether acrylate-*co*-acryloylmorpholine) network with densely interlaced polymer chains. Adapted with permission.^91^ Copyright 2024, Elsevier.

### 5.3 Branched Polymers

Excellent mechanical properties in the polymer require usually the presence of macromolecules with high molecular weight, resulting in entanglements.^91^ At room temperature, chain interdiffusion in most polymers is too slow to heal a damaged area, resulting in poor healing efficacy. To address this issue, researchers have examined the influence of dangling chains in branched polyetherimides on healing kinetics in the absence of noncovalent interactions.^92^ Polyimides were prepared by a two-step polymerization, involving the use of aromatic dianhydride and a fatty dimer diamine. In the first step, polyamic acid was synthesized via a polycondensation reaction between 4,4′-oxidiphthalic anhydride and a branched aliphatic fatty dimer diamine. Subsequently, polyamic acid underwent imidization to form the final polyimide. Four polymers were synthesized by using varying molar ratios of aromatic dianhydride to fatty dimer diamine: D-0.9, D-1.0, D-1.1, and D-1.2 (Figure 7a). These ratios ranged from a 10 mol % excess of aromatic dianhydride to a 20 mol % excess of fatty dimer diamine. Damaged samples with D-1.0 and D-1.1 diamine-to-dianhydride ratios exhibited nearly 100% healing efficiency. Notably, D-1.1 achieved recovering an initial tensile strength of ∼6 MPa within 5 days at 23 ± 2 °C, whereas D-1.0 required 11 days to recover a tensile strength of ∼5 MPa, indicating that a higher diamine-to-dianhydride ratio enhances healing kinetics. The presence of long aliphatic branches with saturated C36 isomers facilitates the healing mechanism by forming a transient supramolecular network through weak van der Waals interactions between the dangling aliphatic chains. This network created a 2D interface promoting adhesion between the damaged surfaces. Subsequently, chain interdiffusion led to the development of a 3D interphase, restoring the mechanical properties of the polymers (Figure 7b). However, samples with lower (D-0.9) and higher (D-1.2) branched dimer diamine contents exhibited no healing after 11 days because of restricted polymer chain mobility. The study showed slow healing kinetics, which are unsuitable for real-time application. To tune the healing time, researchers have investigated the role of branching in the relaxation dynamic of polyurethanes with a high density of hydrogen bonds.^93^ Brush polyurethanes with dangling aliphatic chains with various lengths were synthesized by the polyaddition of hexamethylene diisocyanate (HDI) and several branched diols: 2,2-dibutylpropane-1,3-diol (C4DA), 2,2-diheptylpropane-1,3-diol (C7DA), and 2,2-dioctylpropane-1,3-diol (C8DA), or 1,4-butanediol (BDO) as nonbranched diol (Figure 7c). At 36 °C, the fractured HDI-C7DA and HDI-C8DA exhibited a healing efficiency comparable to HDI-C4DA, recovering 60–70% of the original tensile strength (6–8 MPa from an initial 10.5 MPa). However, a much shorter healing time was necessary for HDI-C7DA and HDI-C8DA (3 h) than for HDI-C4DA (100 h), while HDI-BDO could not be healed after 100 h. The side aliphatic brushes played a crucial role in controlling the chain dynamics. The short side chains prevented crystallization and facilitated main-chain interdiffusion, which in turn increased self-healing kinetics.

**Figure 7:**
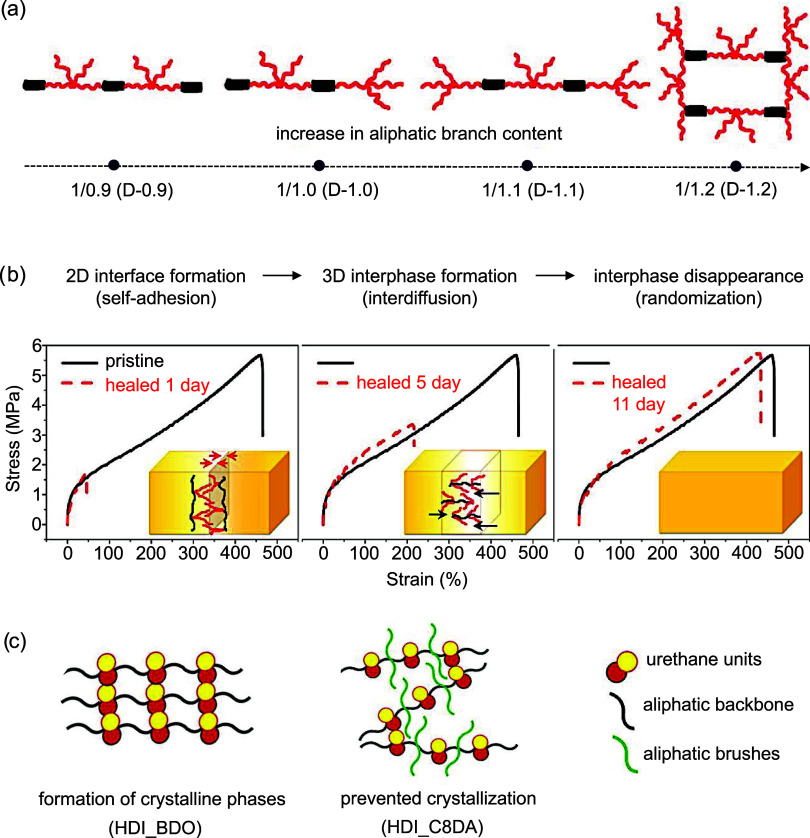
(a) Schematic illustration showing the influence of molar ratio between aromatic dianhydride and fatty dimer diamine on the architecture of polyetherimide. (b) Stress–strain curves showing the healing behavior of poly­(ether imide) at room temperature after 1, 5, and 11 days. Inset: schematic illustrating the three stages of the polyetherimide healing process. Adapted with permission.^92^ Copyright 2016, American Chemical Society. (c) Schematic representations of the macromolecular architecture of polyurethanes without aliphatic branches (HDI_BDO) and with aliphatic branches (HDI_C8DA). Adapted under the terms of the CC-BY-NC-ND license.^93^ Copyright 2019, The Authors, published by American Chemical Society.

Healing time can be further shortened for polymer displaying higher mechanical properties by utilizing glassy polymers with hyperbranched structures.^94^ The hyperbranched molecules were synthesized via nucleophilic substitution between thiocarbonyl diimidazole and bis­(hexamethylene)­triamine, forming thiourea linkages. Subsequently, hyperbranched skeleton terminal groups were tailored with diethanolamine and methyl isothiocyanate to generate methylthiocarbamate groups (Figure 8a). The damaged polymer exhibited healing efficiencies of 64% with a tensile strength of 9.8 MPa and 98% with a tensile strength of 14.9 MPa, relative to the initial tensile strength of 15.3 MPa, under a normal force of 40 N after 1 h at 25 and 30 °C, respectively. Ethanol was found to promote rapid healing of the damaged samples in the absence of external force. An ethanol content of 12–14 mg mm^–2^ applied to the damaged area enabled recovery of a tensile strength of 5.9 MPa within 1 min, with Young’s modulus restored to more than 80% of the original value. The effective self-healing behavior of the polymer network was attributed to its hyperbranched architecture, high density, and mobility of terminal functional groups, as well as dynamic exchange of hydrogen bonds. However, the healing ability of the polymer still depends on either mechanical pressure or solvent assistance, which limits its applicability in environments where such external stimuli are impractical. To address these limitations, another study introduced a new class of randomly hyperbranched polymers that can autonomously self-heal without the need for external triggers.^95^ The hyperbranched polymer was synthesized by Michael addition of *N*,*N*′-methylene diacrylamide and 1,4-butanediamine so that the polymer contained amide groups and secondary amines on the branched units and primary amines as end groups. Self-healing after mechanical damage, demonstrated by a partial recovery of 33%, with a tensile strength of 4.3 MPa out of an initial ∼13 MPa, was achieved within 1 min at 25 °C (Figure 8b). The healing efficiency increased over time, reaching 76% with a tensile strength of 9.9 MPa after 48 h. Fast healing was attributed to the highly mobile branched units and end-groups as well as the hindrance of ordered packing of molecular chains. Moreover, the branched chains could interpenetrate across the fractured surface to form hydrogen bonds. To investigate self-healing polymers with a high tensile strength of 36 MPa, a recent study proposed a novel hyperbranched epoxy vitrimer.^96^ Vitrimers are cross-linked polymers that combine the structural stability of thermosets with the reprocessability of thermoplastics, enabled by dynamic covalent bonds capable of reversible exchange. The vitrimer was prepared via solvent-free polymerization of bis­(2,3-epoxypropyl) cyclohex-4-ene-1,2-dicarboxylate monomer and a phosphorus/silicon-containing polyethylenimine-based curing agent. The polyethylenimine-based curing agent was synthesized from dibenzo­[c,e]­[1,2]­oxaphosphinic acid, polyethylenimine, and dichlorodiphenylsilane. Notches on the material surface (∼110 μm wide), holes (∼10 μm wide), and scratches on the surface completely healed within 12 h at room temperature. Upon partial cut damage, the tensile strength of the vitrimer decreased from 36 MPa to 11 MPa. Healing of the partially cut sample resulted in a recovered tensile strength of ∼35 MPa after 12 h at room temperature, demonstrating a healing efficiency of ∼96%. The self-healing ability of the vitrimers was attributed to the diffusion of hyperbranched chains to the interface at room temperature, which facilitated the reformation of dynamic hydrogen bonds and π–π stacking interactions (Figure 8c). These interactions enabled the polymer chains to rebond, effectively healing the damaged material without the need for external stimuli. To enable self-healing in glassy polymers at −20 °C, a supramolecular nanocomposite was designed using a polymerizable deep eutectic solvent reinforced with polyphenol nanoassemblies.^97^ The photopolymerizable deep eutectic solvent comprised acrylic acid, maleic anhydride, and choline chloride, with poly­(ethylene glycol) diacrylate serving as cross-linker. To construct a dynamic bonding network, ellagic acid nanospheres were incorporated into the polymerizable deep eutectic solvent mixture prior to polymerization. These ellagic acid nanospheres were formed using ultrasonic degradation of tannic acid followed by Fe^3+^-induced coordination, yielding uniform spherical particles of ∼60–140 nm in diameter. The ellagic acid nanospheres are rich in phenolic groups that interact with the carboxyl functionalities of the polymerizable deep eutectic solvent matrix through hydrogen bonding and metal–ligand coordination. The resulting nanocomposite was subsequently polymerized by ultraviolet-induced free-radical polymerization, forming a cross-linked supramolecular network. The fully cut nanocomposite containing 0.04 wt % ellagic acid nanospheres recovered ∼86% of its tensile strength after 6 h at −20 °C, and ∼87% after 1 h at 25 °C, relative to the original strength of 30.6 MPa, without external pressure or stimuli (Figure 8d). The healing mechanism was attributed to rapid secondary relaxations of branched units or carboxyl groups on the backbone of the polymerizable deep eutectic solvent and hydroxy terminal groups of ellagic acid nanospheres, driven by β-, γ-, and δ-relaxation processes with low activation energies ranging from 14.7 to 51.3 kJ mol^–1^ (Figure 8d). These dynamic exchanges are active even below the glass transition temperature of ∼35.8 °C, enabling effective healing in a glassy state. This strategy demonstrates a robust approach for designing mechanically strong, subzero-healable materials.

**Figure 8:**
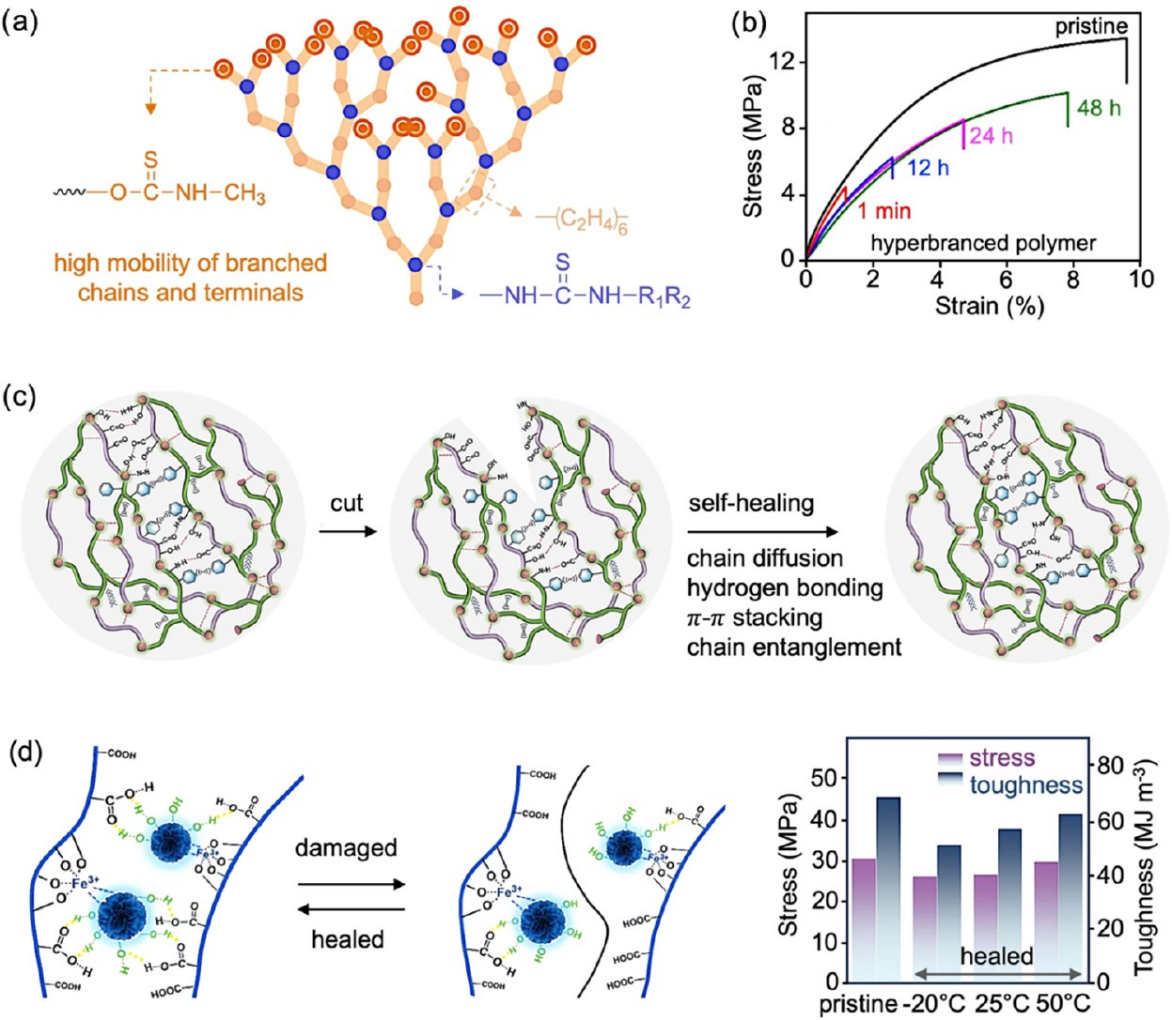
(a) Schematic structure of a hyperbranched polymer. Adapted with permission.^94^ Copyright 2024, Royal Society of Chemistry. (b) Tensile test curves of the pristine and healed hyperbranched polymers at 25 °C with different healing times. Reproduced under the terms of the CC-BY license.^98^ Copyright 2020, The Authors, published by PNAS. (c) Schematic illustration of the self-healing mechanism of a vitrimer. Reproduced under the terms of the CC-BY Creative Commons Attribution 4.0 International License.^96^ Copyright 2024, The Authors, published by Elsevier. (d) Schematic illustration of the self-healing mechanism, showing reversible hydrogen bonding and metal–ligand coordination between carboxyl groups on the polymer chains and phenolic groups on the ellagic acid nanospheres, enabling network reconstruction (Left). (Right) Comparison of tensile strength and toughness of photopolymerizable deep eutectic solvent matrix containing 0.04 wt % ellagic acid nanospheres: pristine and self-healed sample after healing at −20 °C for 6 h, and at 25 and 50 °C for 1 h. Reproduced under terms of the CC-BY license.^97^ Copyright 2023, The Authors, published by Springer Nature.

To facilitate a clearer comparison across different self-healing polymers and to identify effective design, we compile a quantitative summary of representative examples. Table 2 presents a benchmarking matrix that includes key parameters for each system: polymer backbone type, dynamic bonding motif, glass transition temperature, stress and strain at break, healing temperature, healing time, and healing efficiency used. This comparative overview enables the identification of structure–property-function relationships and offers practical guidance for the rational design of next-generation healable polymers.

**Table 2:** Type of backbone, dynamic motif, glass transition temperature (*T*_g_), stress/strain at break (σ/ε), healing temperature, healing time, and healing efficiency for polymers with self-healing at low/room temperatures^a^

backbone	dynamic motif	*T*_g_ (°C)	σ and ε at break	healing *T* (°C)	healing time	healing efficiency (%)	ref
PEGMA-*co*-HEMA-UPy	H-bonding	n.a.	σ = 0.72 MPa	RT	5 min	∼100	^84^
			ε ≥ 300%				
poly(AM-*co*-MPEG)	H-bonding with DES	13.5	σ = 0.24 MPa	RT and −25	30 s and 10 min	∼90	^83^
			ε = 1120%				
poly(MAA-*co*-OEGMA)	H-bonding, imine bonds	∼22	σ = 3.0 MPa	RT	8 min	∼100	^85^
			ε = 520%				
poly(MAA-*co*-OEGMA)-BIFA	H-bonding, π–π	n.a.	σ = 3.7 MPa	35	8 min	100	^86^
			ε = 353%				
poly(etherimide) with fatty dimer diamine	chain interdiffusion	13	σ ∼ 6 MPa	25	11 days	100	^92^
			ε ∼ 470%				
brushed poly(urethane)	H-bonding	49	σ = 10.5 MPa	36	3 h	69	^93^
			ε = 660%				
poly(ACMO-*co*-mPEG480)	H-bonding	n.a.	σ ∼ 8.4 MPa	RT	10 s	86	^88^
poly(ACMO-*co*-mPEG)	H-bonding + Zn^2+^ coordination	47.6	σ = 12.7 MPa	RT	30 s	95	^90^
			ε = 250%				
poly(ACMO-*co*-MPEG480)	H-bonding	47.5	σ = 8.4 MPa	RT	10 s	93	^89^
			ε = 780%				
PMMA/PEGA copolymer	Li^+^-O ion-dipole, PMMA aggregation	n.a.	σ = 18 MPa	24	24 h	98	^82^
			ε = 30,000%				
BHMT/TCDI hyperbranched polymer with thiocarbamate	H-bonding	62.5	σ = 15.3 MPa	30	1 h	98	^94^
			ε ∼ 2.8%				
MBA/BDA hyperbranched polymer	H-bonding	37.0	σ ∼ 13 MPa	25	48 h	76	^98^
			ε ∼ 9%				
PDES with polyphenol nanoassemblies	H-bonding, metal coordination	35.8	σ ∼ 30.6 MPa	–20	6 h	86	^97^
			ε ∼ 300%				
DCNC/PEDA hyperbranched epoxy vitrimer	β-hydroxy ester, H-bonding, π–π	52.1	σ = 36 MPa	25	12 h	96	^96^
			ε = 51.3%				

a Abbreviations: ACMO, acryloylmorpholine; AM, acrylamide; BDA, 1,4-butanediamine; BHMT, bis­(hexamethylenetriamine); BIFA, benzyl imine-functionalized anthracene; DCNC, bis­(2,3-epoxypropyl) cyclohex-4-ene-1,2-dicarboxylate; DES, deep eutectic solvent; EGA, di­(ethylene glycol) methyl ether acrylate; HEMA: 2-hydroxyethyl methacrylate; MAA, methacrylic acid; MBA, *N,N*′-methylenediacrylamide; MMA, methyl methacrylate; mPEG480, poly­(ethylene glycol) 480 methyl ether acrylate; MPEG, poly­(ethylene glycol) methyl ether methacrylate; OEGMA, oligo­(ethylene glycol) methacrylate; PEGMA, poly­(ethylene glycol) methyl ether methacrylate; PEDA, phosphorus/silicon-containing polyethylenimine; TCDI, 1,1-thiocarbonyldiimidazole; UPy, 2-ureido-4­[1*H*]-pyrimidinone.

## 6 Healing by Shape Memory Effect

An ultimate goal for polymer chemists is to fabricate high-strength and high-stiffness polymers with self-healing ability at low temperatures without external stimuli. Viscoelastic length transitions describe temperature-dependent dimensional changes in polymers occurring near *T*_g_, attributed to their inherent viscoelastic behavior. These transitions are typically examined using dynamic mechanical analysis, particularly in shape memory cross-linked polyurethanes.^99^ At *T*_g_, viscous components of the network were responsible for the polymer materials extension upon stretching while retraction occurred due to the stored conformational entropy resulting from chemical or physical cross-linking and chain entanglements. Extensions and retractions can be quantified in terms of the stored entropic energy density Δ*S*_S_, which is a function of the stress at maximum strain σ_SF at ε_max__ and the maximum strain ε_max_ for polymer materials healed after damage:^99^

$$\Delta S_{S}=5.2819\left(\varepsilon_{\max}\right)\left(\sigma_{\mathrm{SF}\;\mathrm{at}\;\varepsilon_{\max}}\right)$$

Δ*S*_S_ can be calculated from the determination of tan δ_max_ measured by dynamic mechanical analysis, corresponding to the maximum of the ratio of loss modulus to storage modulus, and the junction density ν_j_, which is the density of the polymer divided by the average molecular weight between cross-links or entanglements:^99^

$$\Delta S_{S}=1.4011\frac{\left(\tan\delta_{\max}^{2}\right)\left(\nu_{j}^{0.6613}\right)}{\ln\left(\nu_{j}\right)}$$

Thus, the recovery of mechanical properties increases with higher molecular weights due to the presence of more entanglements, enabling storage of conformational entropic energy (Figure 9a). On the contrary, mechanical recovery and energy storage after deformation or damage are limited by chain slippage and flow in low-molecular-weight polymers. Hornat et al. developed mechanically robust polyurethane fibers that are capable of autonomous self-repairing at room temperature.^100^ Polyurethanes with *M*_w_ ∼ 72,000 g mol^–1^or 45,000 g mol^–1^ were synthesized by polyaddition of isophorone diisocyanate and polytetrahydrofuran (*M*_n_ = 250 g mol^–1^). The fibers were partially cut by a razor blade in the direction perpendicular to the fiber axis and left for healing at 25 °C and ∼50% relative humidity. Although fibers fabricated with the higher-molecular-weight polymer were healed after 40 min, the fibers with the low-molecular-weight polymer were not healed (Figure 9b). The damaged fiber with *M*_w_ ∼ 72,000 g mol^–1^ fully recovered, showing a tensile strength and modulus of 21 and 299 mN/tex, respectively, after 40 min at 25 °C. In contrast, the fiber with *M*_w_ ∼ 45,000 g mol^–1^ recovered ∼63% of its tensile strength (7.55 out of 11.90 mN/tex) and ∼89% of its modulus (237 out of 267 mN/tex). Self-healing of the fibers was induced by a decrease of the number of H-bonds in the damaged area and a change of chain conformation resulting from deformation, creating a restoring force responsible for shape recovery (Figure 9c). The healing of the fiber was attributed to the efficient storage (Δ*S*_S_) and release (Δ*S*_R_) conformational entropic energy (Δ*S*_S_ ∼ 31 kJ m^–3^ and Δ*S*_R_ ∼ 27 kJ m^–3^), which enabled a viscoelastic shape memory effect. In contrast, the fiber from the lower-molecular-weight polymer exhibited a significant difference between Δ*S*_S_ (27 kJ m^–3^) and Δ*S*_R_ (17 kJ m^–3^) due to chain slippage. Consequently, fibers produced with the low-molecular-weight polymer were unable to display the shape memory property.

**Figure 9:**
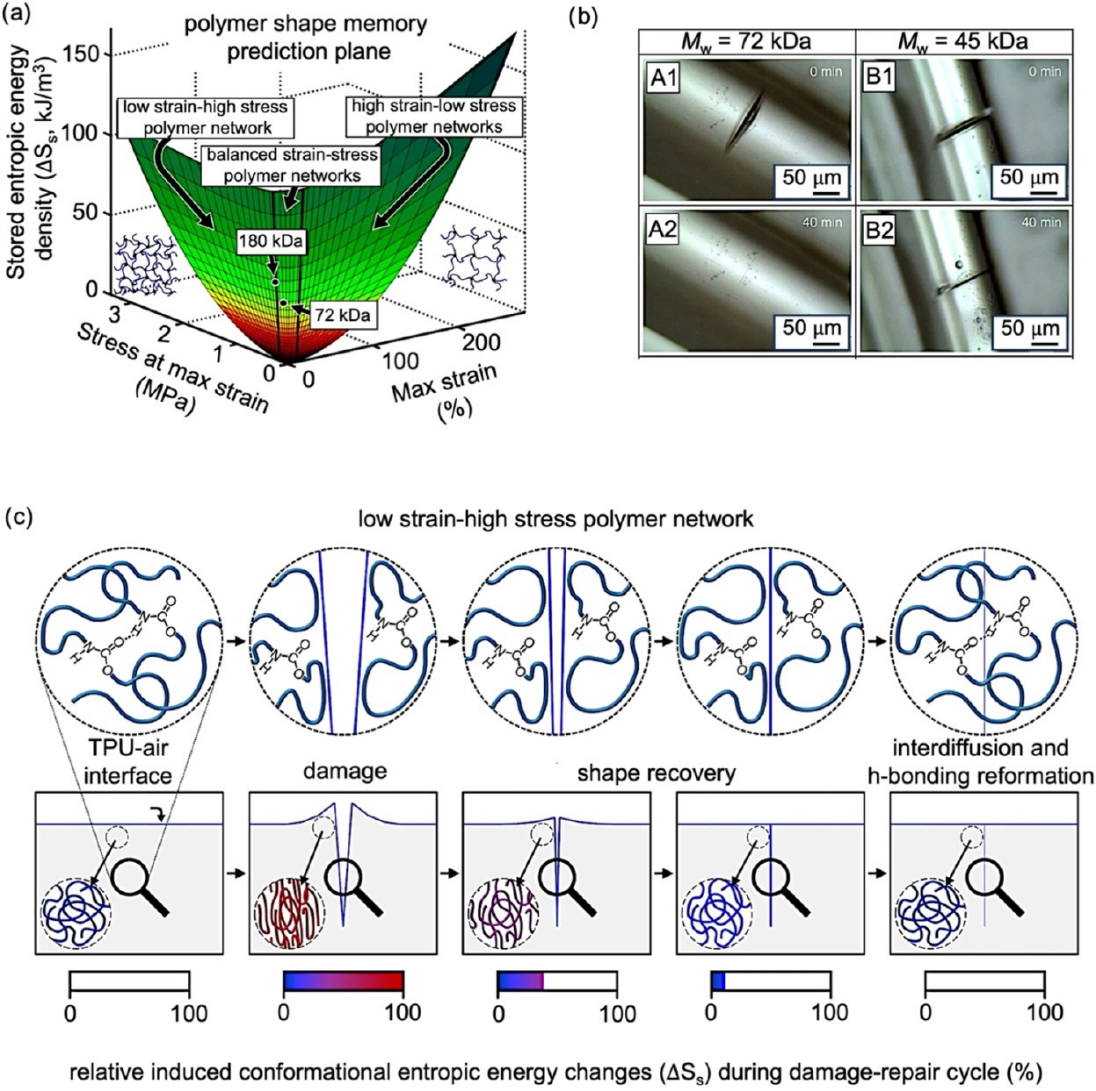
Shape-memory behavior in thermoplastic polyurethane fibers arising from entropy-driven elastic recovery, where deformation stores entropic energy, which is subsequently released to promote crack closure. (a) Shape memory prediction plane for thermoplastic polyurethanes with molecular weights of 180 and 72 kDa. Stored entropic energy density (Δ*S*_S_) is plotted as a function of maximum strain and stress at maximum strain derived from dynamic mechanical analysis (DMA). These viscoelastic length transitions, occurring near the glass transition temperature, reflect the interplay between viscous deformation and elastic recovery. Conformation entropy, resulting from chain entanglements and cross-linking, drives macroscopic retraction upon unloading. (b) Optical images of damaged fibers produced with polymers of *M*_w_ = 72 kDa (A1–A2) or 45 kDa (B1–B2), immediately after mechanical damage (A1 and B1), or after healing for 40 min at 25 °C and ∼50% relative humidity (A2 and B2), showing crack closure. (c) Scheme showing the molecular and macroscopic states in the polymer material during entropy-driven self-healing, induced by chain conformational changes around the damage, disentanglement, and re-entanglement of polymer chains. Reproduced under terms of the CC-BY license.^100^ Copyright 2020, The Authors, published by Springer Nature.

Unlike molecular weight-governed self-healing observed in thermoplastic polyurethanes, this alternative healing strategy utilizes soft Oleogel-based viscoelastic microparticles embedded in a rigid matrix.^101^ The resulting composite enables room-temperature self-healing through structural phase separation and viscoelastic flow. Viscoelastic Oleogel microparticles were synthesized by assembling calcium ions (Ca^2+^) with phosphotungstic acid (H_3_PW_12_O_40_·*x*H_2_O, PTA^3–^), coordinated with oleylamine (OA) and its protonated form (OA^+^). The material formed a physically cross-linked calcium-polyoxometalate network through coordination bonding and electrostatic interactions (Figure 10a). This calcium–polyoxometalate network was dispersed into hydrogenated poly­(1-decene), yielding a chemically stable hydrophobic Oleogel. The resulting Oleogel particles were then incorporated into a commercial waterborne epoxy, creating a phase-separated composite with a soft–hard hybrid architecture. Coatings with a thickness of 65 μm containing 40 wt % Oleogel particles were fabricated onto carbon steel panels, followed by scratching. The coatings demonstrated a repeatable healing performance, with an impedance modulus (|*Z*|_0.01 Hz_) recovering above 10^10^ Ω·cm^2^ after multiple damage-healing cycles (Figure 10b). Healing occurred via two synergistic processes: entropy-driven shape recovery of the particles, leading to rapid crack narrowing within 10 min, and surface-tension-driven Oleogel flow, fully restoring barrier protection after 5 h at room temperature (Figure 10c). However, as the healing mechanism was driven by particles flow rather than polymer chain entanglement recovery, mechanical restoration at the crack site relied on external stimulation such as ethanol spraying.

**Figure 10:**
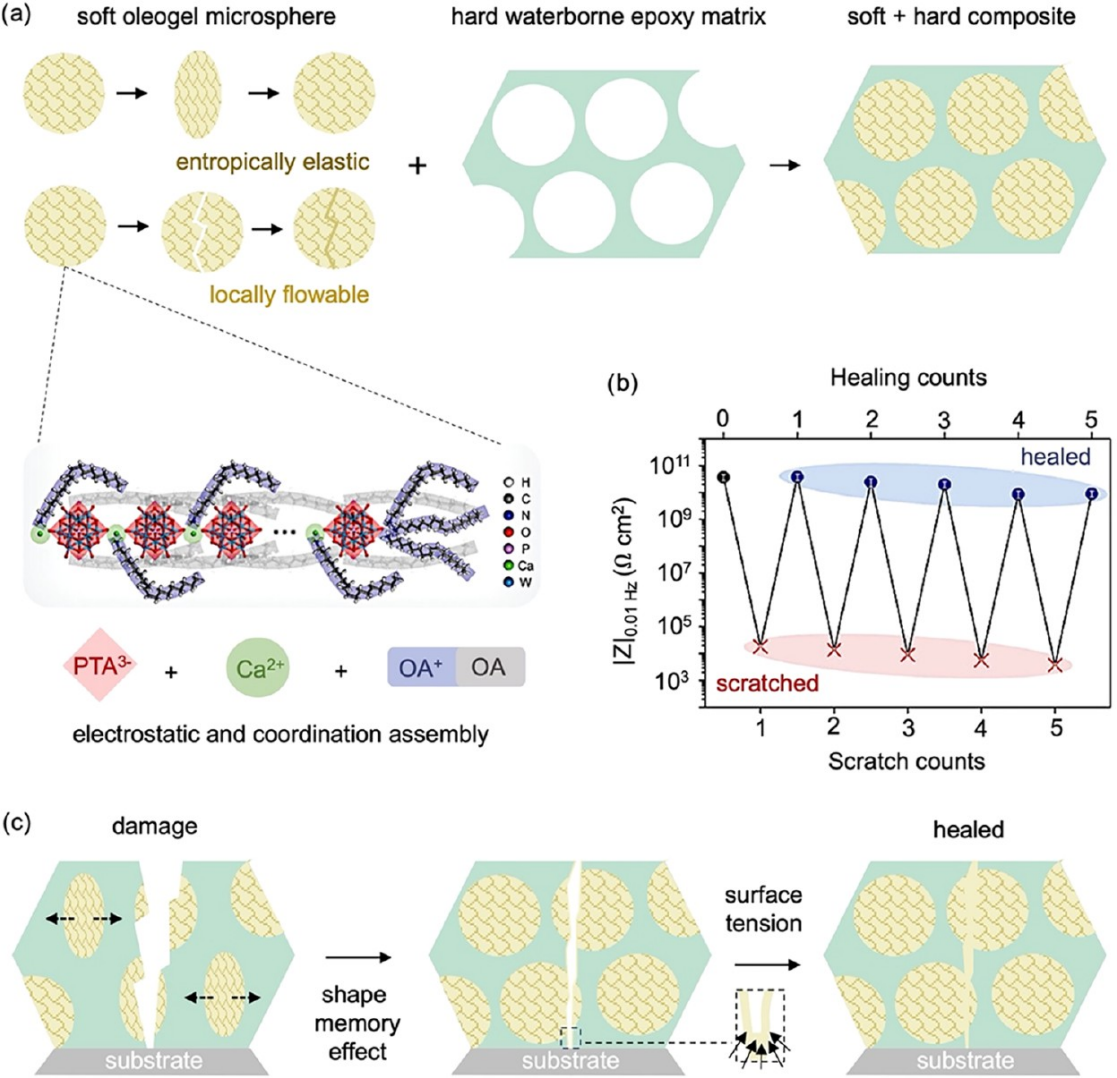
(a) Schematics showing Oleogel-based self-healing. (b) Impedance modulus (|*Z*|_0.01 Hz_) of epoxy containing the Oleogel after repeated scratching and healing. (c) Schematic representation of the entropy- and surface-tension-driven scratch healing and reformation of van der Waals interactions, coordination, or electrostatic interactions. Adapted with permission.^101^ Copyright 2024, Wiley.

## 7 Healing with Assistance of External Stimuli

### 7.1 Healing with Solvent Assistance

Mechanically robust polymers, especially thermosetting polymers, need external stimuli to facilitate molecular mobility. Self-healing of polymers can be triggered by the presence of a solvent or swelling agent. The mechanical strength of polymers can be enhanced during healing by initial wetting of the surface of the damaged area driven by van der Waals forces and subsequently interdiffusion of polymer chains.^102^ Besides, swelling of an epoxy vitrimer with a solvent was found to activate bond exchange reactions.^103^ The epoxy vitrimer was prepared by the reaction between bisphenol A diglycidyl ether with adipic acid and triazobicyclodecene as catalyst, which was covalently connected to the network (Figure 11a). At room temperature, transesterification did not occur in the absence of a catalyst. In contrast, the mechanically damaged vitrimer prepared with the catalyst was repaired after dropping THF onto the damaged area. The welded sample exhibited almost the same mechanical properties as for the pristine sample (∼20 MPa stress), according to lap-shear tests (Figure 11b). In order to avoid the assistance of an operator to add solvent for repairing the polymer matrix, the solvent can be entrapped in capsules that are then embedded in the matrix to repair. The healing of cracks in epoxy was achieved with urea-formaldehyde microcapsules with an average diameter of 160 ± 20 μm entrapping chlorobenzene, xylene, or hexane.^18^ Cracks in epoxy thermosets were repaired using an encapsulated chlorobenzene system at 22 °C within 24 h, resulting in 82% healing efficiency with a load of ∼98 N compared to the original value of ∼120 N, as calculated from fracture peak loads. This healing efficiency was comparable to the value (78%) obtained for cracked epoxy without capsules that were manually treated with chlorobenzene. Low healing efficiencies of 38% and 0% were obtained with epoxy embedding capsules, which contained xylene and hexane, respectively. The higher healing efficiency measured with capsules containing chlorobenzene was attributed to the polarity of this solvent, which allowed for plasticization of the epoxy matrix and, hence, molecular mobility. However, swelling may affect the mechanical properties of the polymer materials. Moreover, some organic solvents are toxic, and most of them are highly flammable.

**Figure 11:**
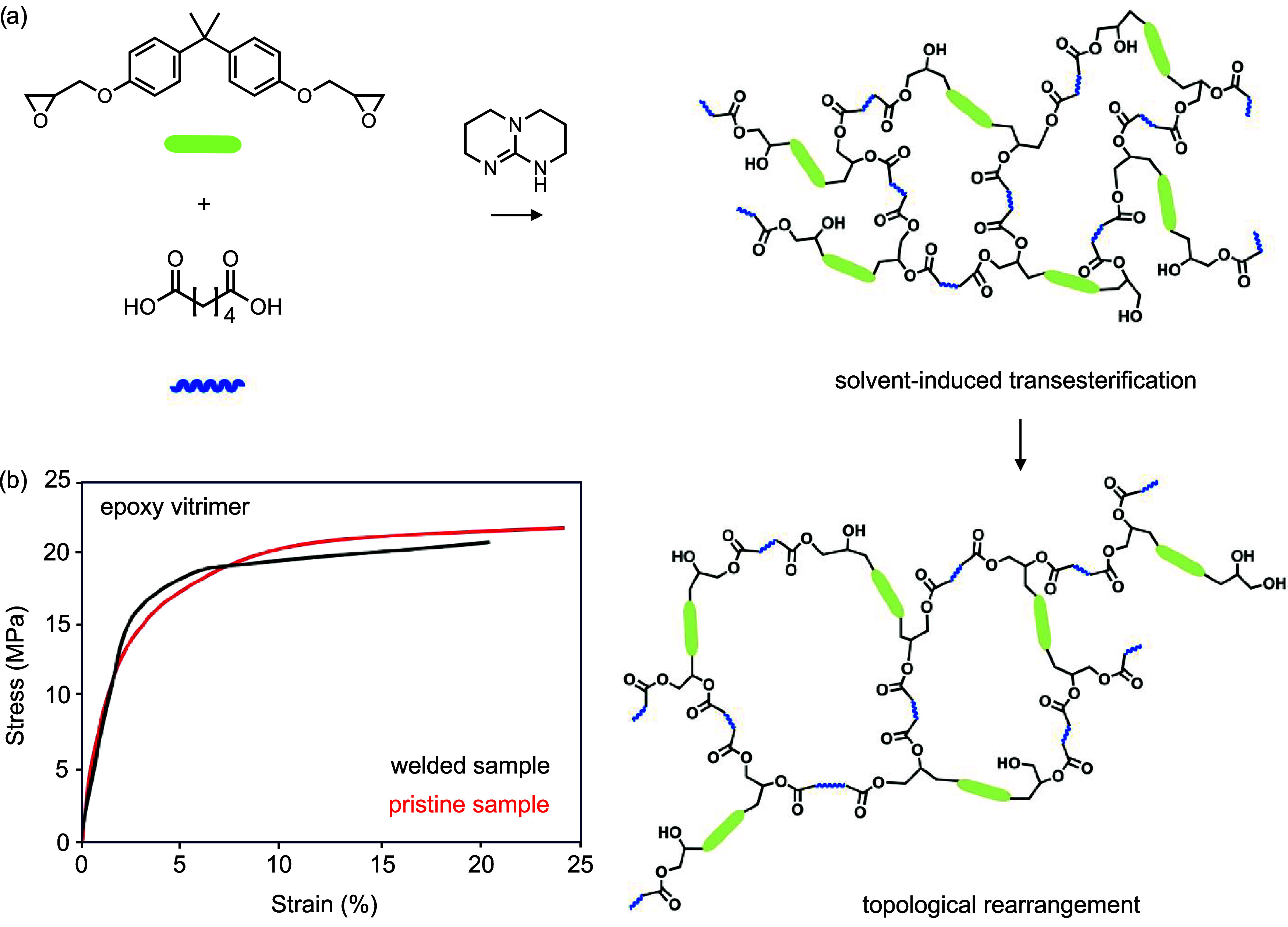
(a) Scheme showing the synthesis of an epoxy vitrimer and transesterification after swelling of the epoxy with a solvent. (b) Stress–strain curve obtained from lap shear measurement of welded and pristine epoxy vitrimers at a ramp force of 0.5 N/min. Reproduced under terms of the CC-BY license.^103^ Copyright 2018, The Authors, published by Springer Nature.

### 7.2 Healing with Water

Water has gained attention as a sustainable and biocompatible trigger for self-healing in polymers because it can significantly enhance healing efficiency. To investigate water-assisted self-healing in polymers with high tensile strength (up to 41 MPa), a polyurea elastomer was synthesized via a multistep approach involving the formation of a segmented polyurea backbone, followed by dynamic covalent cross-linking.^104^ A mixture of short-chain (*M*_n_ ∼ 400 g mol^–1^) and longer-chain (*M*_n_ ∼ 2000 g mol^–1^) poly­(propylene glycol) was first reacted with a combination of methylene diphenyl diisocyanate and isophorone diisocyanate to form a urea-linked polymer network (Figure 12a). Subsequently, trimethylolpropane tris­[poly­(propylene glycol)-amine terminated] and terephthalaldehyde were introduced to form dynamic imine cross-links through condensation with amine termini. The resulting elastomer featured a hierarchical network of strong and weak hydrogen bonds in addition to dynamic imine bonds, enabling robust mechanical integrity and room-temperature healing. After the cut surfaces were immersed in water for 1 min, the sample was rejoined and healed at 25 °C under ∼100% relative humidity, resulting in a full restoration of the sample integrity within 72 h and a healing efficiency of ∼92%. The healed material recovered a tensile strength of 38 MPa and an elongation at break of ∼850%. The healing mechanism relied on water-facilitated reformation of imine bonds and hydrogen bond reorganization, which collectively drove chain mobility and interfacial reconnection across the damaged regions. Although healing was relatively slow, this system exhibited remarkable mechanical performance. In another study, polyurethane elastomer was synthesized through a two-step polyaddition between isophorone diisocyanate and poly­(tetramethylene ether glycol).^105^ Dimethylolpropionic acid was added to introduce carboxylic acid groups. This prepolymer was subsequently extended by using *N,N*-bis­(2-hydroxyethyl)-3-aminopropenamide. The ionic sites, provided by the tertiary amine and carboxylic acid, were designed to form electrostatic interactions post-neutralization, while abundant hydrogen bonding arose from urethane linkages and hydroxyl groups. A fully cut sample was healed in the presence of water at room temperature for 36 h, achieving ∼105% healing efficiency with a tensile strength of ∼13 MPa, slightly exceeding the tensile strength of the original material. The healing mechanism was attributed to water-induced ionization, which enhanced chain mobility and facilitated the reformation of electrostatic and hydrogen bonds at the damaged interface, enabling an efficient autonomous repair under mild conditions (Figure 12b). To investigate ultrafast healing polymers at low temperature, the multifunctional polyurethane was synthesized through a stepwise process designed to incorporate both strong mechanical properties and water-assisted self-healing capability.^106^ The prepolymer was prepared by reacting poly­(ethylene glycol) (*M*_n_ ∼ 6000 g mol^–1^) with hexamethylene diisocyanate in the presence of a catalyst. This intermediate was then extended using 5,5′-diamino-2,2′-bipyridine, introducing pyridine moieties capable of coordinating with europium ions (Eu^3+^). The final coordination step, involving EuCl_3_·6H_2_O, resulted in a highly cross-linked polymer network through Eu^3+^–pyridine interactions. This polyurethane exhibited a low-temperature self-healing behavior when it was exposed to water. At 4 °C, surface cracks disappeared within 3 min following the application of a thin water layer, with the healed material achieving a tensile strength of ∼47 MPa and a healing efficiency of 96% (Figure 12c,d). Water-assisted healing enabled full recovery through enhanced molecular mobility and hydrogen bonding of the poly­(ethylene glycol) segments, supported by reversible reformation of coordination bonds. The synergistic contributions of hydrogen bonding, coordination cross-links, and the high crystallinity of poly­(ethylene glycol) provided both mechanical durability and efficient healing under low temperatures.^106^ Crystalline domains act as physical cross-links that stabilize the dimensional structure of material and enhance mechanical strength. However, they can simultaneously hinder healing by restricting chain diffusion at the damaged interface. In this system, no transient melting of the crystalline regions occurs during repair. Instead, water promotes localized chain mobility at the damaged interface through hydrogen bonding, facilitating interfacial healing without disrupting the bulk crystalline structure, thereby enabling effective self-healing at 4 °C. Notably, the systems reported by Wang et al.^96,106^ both exhibit >95% recovery with tensile strengths ranging from ∼35 to 47 MPa, effectively addressing the trade-off between chain mobility and mechanical robustness.

**Figure 12:**
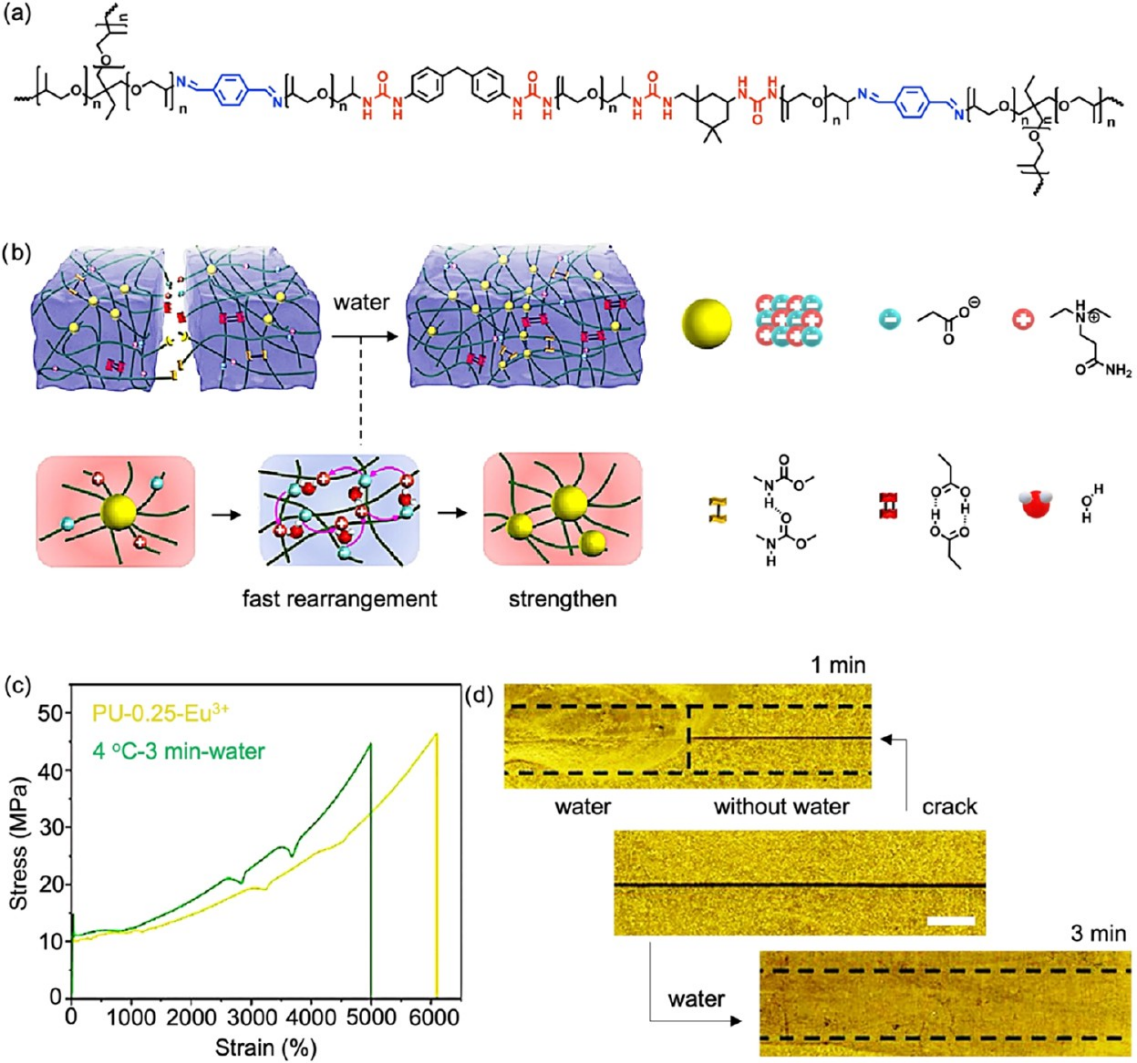
(a) Chemical structure of poly­(propylene glycol)-based poly­(urea-imine). Adapted with permission.^104^ Copyright 2020, American Chemical Society. (b) Schematic illustration of the molecular behavior during the self-healing process of poly­(tetramethylene ether glycol)-based polyurethane containing ionic aggregation, driven by the rearrangement of hydrogen bonds and ionic interactions in the presence of water. Adapted with permission.^105^ Copyright 2024, Elsevier. (c) Stress–strain curve of original poly­(ethylene glycol)-based-polyurethane containing 0.25 molar ratio of Eu^3+^ and after healing for 3 min at 4 °C with water. (d) Photographs of poly­(ethylene glycol)-based poly­(urethane) containing 0.25 molar ratio of Eu^3+^, showing a crack and the healed samples after treatment at 4 °C with water for 1 and 3 min. Scale bar: 1 mm. Adapted with permission.^106^ Copyright 2025, Wiley.

Water- or solvent-triggered self-healing materials operating at low or room temperature offer improved healing efficiency but also face challenges related to mechanical stability and environmental constraints. These stimuli-responsive materials often benefit from enhanced chain mobility, plasticization, or dynamic bond reformation facilitated by the presence of water or organic solvents. The addition of external stimuli can result in rapid healing under low or room temperatures and can be advantageous for large-scale or open-environment applications where humidity or moisture is naturally present. However, these systems also present notable limitations. Localized solvent uptake may lead to inhomogeneous healing, mechanical softening, or plastic deformation near the damaged interface, compromising the long-term mechanical integrity of the material. Additionally, the requirement for water or solvent restricts their applicability in sealed, dry, or solvent-incompatible environments. Therefore, although solvent-activated healing can be highly efficient in controlled or humid conditions, careful consideration of environmental compatibility, mechanical performance retention, and long-term stability is critical for practical deployment.

## 8 Conclusions and Outlook

Low-temperature self-healing polymers represent a promising class of materials capable of restoring functionality without the need for elevated temperatures. Highly mobile polymers, particularly those with low glass transition temperatures such as polydimethylsiloxanes containing dynamic noncovalent and covalent bonds, exhibit autonomous healing at room and subzero temperatures through chain diffusion and interfacial energy minimization. This behavior can be further enhanced by incorporating flexible segments or branching units, which disrupt chain packing and increase the free volume, thereby facilitating chain mobility at the damage interface. While these systems offer excellent room-temperature healing, their healing performance often depends on achieving a delicate balance between mobility and mechanical integrity.

A forward-looking strategy should thus pivot toward engineering supramolecular interactions and dynamic covalent chemistries operating under mild conditions. The key lies in maximizing interfacial chain mobility without external heat: introducing flexible, low glass transition temperature domains or plasticizing segments can facilitate diffusion under ambient conditions. Embedding reversible bonding motifs, such as hydrogen bonding, metal–ligand coordination, π–π stacking, or Diels–Alder reactions, can provide both the reversibility and specificity needed for autonomous repair at low temperatures.

In contrast, shape-memory-assisted healing relies on the storage and release of conformational entropic energy to close damage sites. This process is limited for low-molecular-weight polymers due to insufficient entanglement or junction density needed to resist chain slippage during deformation. In such cases, healing primarily occurs through slower surface energy-driven polymer flow, with the rate of repair being closely tied to the cross-link density of material. Excessively cross-link density can impede timely healing, making practical self-repair unfeasible under ambient conditions. Importantly, a balance must be achieved between enabling sufficient molecular mobility for self-healing and maintaining mechanical stability.

External stimuli such as solvents or moisture have been introduced to accelerate healing processes or activate dynamic bonds, offering enhanced control over the healing time and location. However, these strategies present potential limitations, including limited stimulus penetration, risk of material degradation, increased system complexity, environmental concerns, and higher associated costs, all of which may complicate practical deployment. The design of next-generation self-healing polymers will benefit from the combination of these strategies. Hybrid systems that integrate dynamic bonding with shape-memory elements or respond selectively to external cues could offer enhanced healing efficiency while maintaining robust mechanical performance. Additionally, future advances could be driven by the combination of molecular design, nanoscale architectures, and responsiveness to external fields, such as magnetically or electrically induced motion. Integrating multiscale modeling and machine-guided design to predict structure–property relationships will also be essential for optimizing chain dynamics, network connectivity, and healing responses under real-world conditions. Despite these developments, standardizing the evaluation of the self-healing performance remains a key challenge in the field. Indeed, the reported healing efficiencies are often based on varying mechanical properties and testing conditions. Among these, tensile strength recovery is the most commonly used metric because of its direct relevance to structural integrity. However, toughness recovery quantified as the area under the stress–strain curve may offer a more comprehensive assessment, as it simultaneously accounts for both stress and strain, reflecting the ability of the material to absorb and dissipate energy during deformation. Additional parameters, such as strain at break and Young’s modulus, are also frequently reported, with healing efficiency typically calculated as the ratio of the healed property to its pristine or undamaged property. Beyond mechanical properties, many studies assess healing efficiency based on the recovery of specific functionalities relevant to practical applications. These include corrosion protection, electrical conductivity, hydrophobicity, optical transparency, and other performance criteria. While functional metrics are not directly convertible to mechanical values, their inclusion is essential for evaluating application-specific performance and real-world viability. To facilitate meaningful comparison across studies, it is recommended that future work report both mechanical (recovery of stress and strain at break) and functional recovery data.

Ultimately, by synergizing chemistry, physics, and materials engineering, low-temperature self-healing polymers can evolve into scalable, multifunctional systems suited for coatings, soft electronics, and biomedical devices, marking a major step toward resilient and sustainable material technologies.
